# Fortilin binds and stabilizes MEF2C, activates it through phosphorylation, and drives transcription of the cell structural and survival protein CTNNA3

**DOI:** 10.1016/j.jbc.2026.111417

**Published:** 2026-04-01

**Authors:** Mari Nakashima, Sandipan Mukherjee, Decha Pinkaew, Uttariya Pal, Jolanda van Hengel, Geert Berx, Ken Fujise

**Affiliations:** 1 Division of Cardiology, Department of Medicine, University of Washington, Seattle, Washington, USA; 2 Division of Pulmonary, Critical Care, and Sleep Medicine, Department of Medicine, Houston Methodist Hospital, Houston, Texas, USA; 3 Department of Human Structure and Repair, Faculty of Medicine and Health Sciences, Ghent University, Ghent, Belgium; 4 Department of Biomedical Molecular Biology and Cancer Research Institute Ghent (CRIG), Ghent University, Ghent, Belgium; 5 Department of Laboratory Medicine and Pathology, University of Washington, Seattle, Washington, USA

**Keywords:** alpha-T-catenin, CTNNA3, fortilin, HRF, MEF2C, phosphorylation, protein–protein interaction, TCTP, TPT1, transcription

## Abstract

Fortilin, a 172 amino acid modulator protein that positively regulates survival and growth pathways, is one of the most abundantly expressed proteins in the heart, and its loss leads to lethal heart failure. While fortilin binds and protects catenin alpha-3 (CTNNA3)—a cardiomyocyte structural and survival protein—against degradation, its role in the transcriptional regulation of CTNNA3 has remained unknown. Here, we report that fortilin also promotes *CTNNA3* transcription. Mechanistically, we found that fortilin specifically bound to the N-terminal region (amino acids 1–85) of myocyte enhancer factor 2C (MEF2C)—a transcription factor that drives CTNNA3 expression—but not to MEF2A, MEF2B, or MEF2D, as shown by microscale thermophoresis, proximity ligation assay, and *in vitro* and *in vivo* coimmunoprecipitation Western blot analyses. Molecular docking and site-directed mutagenesis identified a critical binding interface involving aspartic acid 25 of fortilin, whereby an aspartic acid 25-to-alanine mutation markedly weakened binding to MEF2C. In addition, fortilin protected MEF2C against ubiquitination and proteasomal degradation and promoted MEF2C serine 59 phosphorylation, a modification essential for its transcriptional activity, both in a binding-dependent manner. Loss of fortilin significantly impaired MEF2C binding to nuclear DNA, reduced *CTNNA3* promoter–driven luciferase activity in an MEF2C-dependent fashion, and lowered RNA polymerase II occupancy on the *CTNNA3* locus. These data suggest that fortilin is a previously unrecognized transcriptional cofactor of MEF2C. By stabilizing MEF2C and promoting its activating phosphorylation, fortilin enhances the transcription of *CTNNA3* while simultaneously stabilizing CTNNA3 protein, thereby sustaining CTNNA3 expression and supporting myocardial structural integrity.

Fortilin—also known as translationally controlled tumor protein, histamine-releasing factor, and TPT1—is a ubiquitously expressed and highly conserved 172 amino acid 20 kDa protein. It was originally cloned in the 1980s as a molecule whose mRNA is sequestered within ribonucleoprotein particles, rendering it inaccessible to the protein-synthesizing machinery of the cell (1, 2, 3). Fortilin is present in the nucleus (4), cytosol (4), and extracellular (5, 6, 7) spaces, where it is involved in diverse cellular processes, including apoptosis inhibition (4, 8, 9, 10, 11, 12, 13, 14), cell cycle progression (15, 16, 17, 18, 19, 20), histamine release during allergic reactions (21, 22), and transforming growth factor beta pathway inhibition (7). It is a modulator protein that exerts its biological activities by binding to an executioner protein and either activating or inhibiting its function. For example, as an antiapoptotic molecule, fortilin binds to antiapoptotic molecules, such as peroxiredoxin-1 (PRX1) (12), myeloid leukemia cell differentiation protein 1 (MCL1) (23, 24), and catenin alpha-3 (CTNNA3) (9), and positively regulates them while binding and negatively regulating proapoptotic molecules, such as inositol-requiring enzyme type 1 (13) and the tumor suppressor protein p53 (TP53) (11).

Fortilin is one of the most abundantly expressed proteins in the heart (25), and its expression is significantly reduced in the myocardium of patients with both ischemic and nonischemic cardiomyopathy compared with healthy controls (14). In mice, fortilin deficiency results in lethal heart failure (14), suggesting a causal relationship between fortilin and the functional integrity of the heart. Although fortilin-deficient cardiomyocytes exhibit elevated levels of the proapoptotic protein p53 and increased apoptosis, mice lacking both fortilin and p53 still develop heart failure, albeit in a milder form, indicating that fortilin supports cardiac function through both p53-dependent and p53-independent pathways (14).

Intriguingly, we recently demonstrated that fortilin specifically binds to CTNNA3 and protects it against phosphorylation-induced ubiquitination and subsequent proteasome degradation (9). CTNNA3 is an 895 amino acid ∼100 kDa protein that is a member of the α-catenin family, which also includes CTNNA1, CTNNA2, and CTNNB (26). CTNNA3 is abundantly expressed in the heart, skeletal muscle, testis, and brain (26). This protein, along with other α-catenins, maintains the structural integrity of the cell by linking the cadherin-based adherens junction complex to the actin cytoskeleton (26). It also protects cells against apoptosis (9). Li *et al.* (27) showed that constitutive *Ctnna3* KO mice developed dilated cardiomyopathy by 3 months of age. Collectively, these observations support the notion that fortilin preserves cardiac function, at least in part, by binding to CTNNA3 and maintaining its appropriate expression through protection from proteasomal degradation. They also suggest that the CTNNA3 pathway may constitute the p53-independent pathway by which fortilin supports myocardial integrity.

While investigating how fortilin maintains CTNNA3 levels in the cell, we found that fortilin not only binds to CTNNA3 and protects it against degradation but also promotes its transcription. This unexpected discovery led us to explore the mechanism by which fortilin may enhance *CTNNA3* transcription. Vanpoucke *et al.* (26) previously reported that *CTNNA3* transcription is directly regulated by myocyte enhancer factor 2C (MEF2C), a key transcriptional factor involved in cardiac development and function. Based on this finding, we hypothesized that fortilin binds to MEF2C and promotes its transcriptional activity. To test this hypothesis, we employed microscale thermophoresis (MST), proximity ligation assay (PLA), and both *in vitro* and *in vivo* coimmunoprecipitation (co-IP) Western blot analyses to define and characterize the fortilin–MEF2C interaction and investigate its biological significance. In addition, based on molecular docking and site-directed mutagenesis, we identified a critical binding interface involving aspartic acid 25 (Asp^25^) of fortilin and the N-terminal region of MEF2C, including threonine 51 (Thr^51^), leading to the generation of an MEF2C-binding–deficient fortilin mutant (fortilin^D25A^). Our results support the conclusion that fortilin functions as a transcriptional cofactor of MEF2C and promotes MEF2C-mediated transcription of the *CTNNA3* gene by protecting MEF2C from proteasomal degradation and maintaining its activating phosphorylation.

## Results

### Fortilin physically interacts with MEF2C

**Figure 1:**
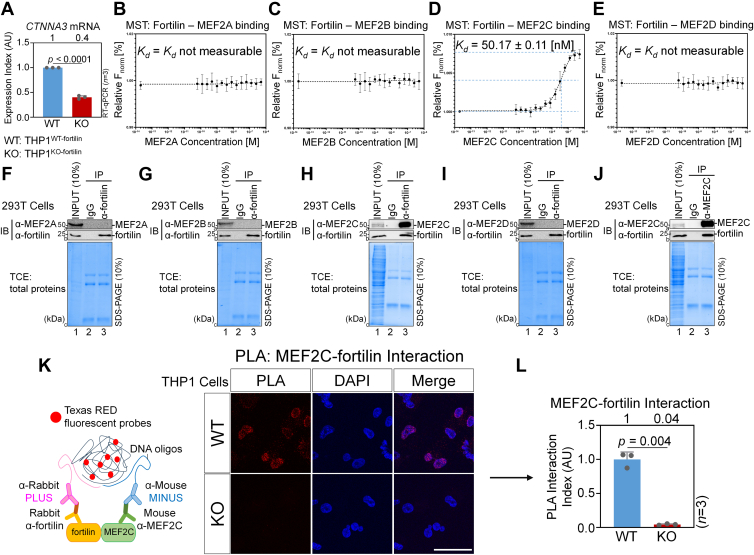
**Fortilin physically binds MEF2C.***A,* quantitative RT–PCR (qRT–PCR) analysis of THP1^WT-fortilin^ and THP1^KO-fortilin^ cells (*n* = 3, *p* < 0.0001, two-sample *t* test). *B*–*E,* MST to characterize the interaction of fortilin with MEF2 family proteins, MEF2A, MEF2B, MEF2C, and MEF2D. *K*_*d*_s were calculated using NanoTemper Analysis software based on the relative F_norm_ values. *F*–*I, in vivo* forward co-IP assays. Fortilin was immunoprecipitated from the TCLs of 293T cells using an α-fortilin Ab. Successful IP of fortilin and co-IP of MEF2C and other MEF2 isoforms were assessed by Western blotting using α-fortilin Ab and Abs against MEF2A, MEF2B, MEF2C, and MEF2D, respectively. Total protein was visualized in the SDS-gel containing TCE. *J, in vivo* reverse co-IP assay. MEF2C was immunoprecipitated from the 293T TCL using an α-MEF2C Ab. Successful IP of MEF2C and co-IP of fortilin were assessed by Western blotting using α-MEF2C and α-fortilin Abs, respectively. Total protein was visualized in an SDS-gel containing TCE. *K* and *L,* PLA to evaluate the fortilin–MEF2C interaction. Phorbol 12-myristate 13-acetate-treated THP1^WT-fortilin^ and THP1^KO-fortilin^ cells cultured on chamber slides were subjected to a standard PLA assay using rabbit α-fortilin and mouse α-MEF2C Abs. Nuclei were counterstained by DAPI (*blue*). *Red* puncta indicated that fortilin and MEF2C were located within 30 nm of each other. The scale bar represents 50 μm. *K,* the PLA interaction index was calculated as the number of PLA puncta per nucleus within a field of view and expressed in AU. Three fields were quantified for each sample, and three independent experiments were performed. Statistical significance was evaluated using a Welch’s two-sample *t* test (*L*). Ab, antibody; AU, arbitrary unit; DAPI, 4′,6-diamidino-2-phenylindole; α-FLAG, anti-FLAG (DYKDDDDK) antibody (Ab); F_norlm_, normalized fluorescence value; α-fortilin, rabbit anti-fortilin monoclonal Ab; Fortilin KO, THP1 cells in which the *fortilin* (*TPT1*) genes have been deleted by the CRISPR–Cas9 technology (THP1^KO-fortilin^); Fortilin WT, THP1 cells expressing WT fortilin (THP1^WT-fortilin^); α-His_6_, anti-hexahistidine Ab; IB, immunoblot; IgG, normal rabbit immunoglobulin G; INPUT, 10% of TCLs used for IP; IP, immunoprecipitation; α-MEF2C, anti-MEF2C Ab; MST, microscale thermophoresis; PLA, proximity ligation assay; TCE, 2,2,2-trichloroethanol; TCL, total cell lysate.

We previously reported that fortilin binds to CTNNA3 and prevents it from being phosphorylated, ubiquitinated, and proteasomally degraded (9). It remained unclear, however, whether fortilin also regulates *CTNNA3* transcription. To address this, we used real-time reverse transcription quantitative PCR (RT–qPCR) to quantify *CTNNA3* mRNA levels in THP1 cells in which the fortilin gene was deleted by CRISPR–Cas9 technology (THP1^KO-fortilin^) and in control WT cells (THP1^WT-fortilin^) (28) (Fig. S1*A*). We found that *CTNNA3* mRNA levels were markedly reduced by approximately 60% in THP1^KO-fortiln^ cells compared with THP1^WT-fortilin^ cells (Fig. 1*A*). We also analyzed two fortilin-dependent genes (*CD68*, *LGALS3* [*MAC2*]) and three fortilin-independent genes (*VWF*, *tissue factor* [*TF*], and *NOS3*), as reported previously (28). The magnitude of *CTNNA3* mRNA reduction in THP1^KO-fortilin^ cells was comparable to that observed for the other fortilin-dependent genes (Fig. S1, *B* and *C*) and clearly distinct from that of the fortilin-independent genes (Fig. S1, *D–F*). These data suggest that fortilin promotes *CTNNA3* transcription.

Fortilin binds to the sequence-specific DNA-binding domain of p53, a tumor suppressor and transcriptional factor, thereby inhibiting p53-mediated transcription of proapoptotic and cell cycle arrest genes (11). In addition, *CTNNA3* transcription is directly regulated by MEF2C, which is a transcriptional factor involved in cardiac development and function (26). Therefore, we used MST to test whether fortilin binds to MEF2 family member proteins (29). MST is a solution-based method used to quantify molecular interactions by detecting molecular movement in a laser-generated temperature gradient (30). We found that fortilin bound to MEF2C with a dissociation constant (*K*_*d*_) of 50.17 ± 0.11 nM, whereas no detectable binding to MEF2A, MEF2B, or MEF2D was observed within the tested concentration range (Fig. 1, *B–E*).

To further validate the direct interaction between fortilin and MEF2C, we performed *in vitro* co-IP Western blot analyses using a reaction mixture containing recombinant FLAG-tagged fortilin (fortilin-FLAG), MEF2C-FLAG, and hexa-histidine–tagged NQO2 (NQO2-His_6_). NQO2 is known to not interact with fortilin (12). We found that anti–fortilin monoclonal antibody (α-fortilin mAb) (Fig. S1*G*, a3), but not mouse immunoglobulin G (IgG) (Fig. S1*G*, a2), immunoprecipitated fortilin-FLAG. In addition, MEF2C-FLAG was coimmunoprecipitated in the presence of fortilin-FLAG (Fig. S1*G*, b3) but not in its absence (Fig. S1*G*, b2). These results suggest that fortilin directly and specifically interacts with MEF2C *in vitro*.

We then conducted *in vivo* experiments to further confirm the specific interaction between fortilin and MEF2C and to determine whether fortilin interacts with other MEF2 isoforms. We subjected the total cell lysates (TCLs) from 293T cells to co-IP Western blot analyses, in which we immunoprecipitated fortilin using α-fortilin mAb and assessed the presence of coimmunoprecipitated MEF2C and other MEF2 isoforms by immunoblotting (Fig. 1, *F–I*). The α-fortilin mAb (Fig. 1, *F–I*, b3), but not control IgG (Fig. 1, *F–I*, b2), efficiently immunoprecipitated endogenous fortilin. Under these conditions, MEF2C coprecipitated only in the presence of fortilin (Fig. 1*H*, a3) but not in its absence (Fig. 1*H*, a2). In contrast, fortilin did not coimmunoprecipitate MEF2A, MEF2B, or MEF2D under identical experimental conditions (Fig. 1, *F*, *G* and *I*). Together, these results demonstrate that fortilin binds to MEF2C in an isoform-specific manner, consistent with the findings from MST assays (Fig. 1, *B–E*).

We also performed reverse co-IP Western blot analyses, in which we immunoprecipitated MEF2C with α-MEF2C mAb and probed for coimmunoprecipitated fortilin (Fig. 1*J*). We found that α-MEF2C mAb (Fig. 1*J*, a3), but not control IgG (Fig. 1*J*, a2), immunoprecipitated MEF2C protein. In the system, fortilin was coimmunoprecipitated only in the presence of MEF2C (Fig. 1*J*, b3) but not in its absence (Fig. 1*J*, b2).

To verify the direct interaction between fortilin and MEF2C *in situ*, we performed a PLA using rabbit α-fortilin mAb and mouse α-MEF2C mAb in THP1 cells in which the fortilin gene was deleted using CRISPR–Cas9 technology (31) (THP1^KO-fortilin^, control = THP1^WT-fortilin^). We previously reported the generation and characterization of THP1^KO-fortilin^ and THP1^WT-fortilin^ cells (9). We observed numerous red fluorescent puncta in the nuclei of THP^WT-fortilin^ cells, indicating close proximity (<30 nm) between fortilin and MEF2C (Fig. 1, *K* and *L*; WT). In contrast, a red signal was not detected in THP1^KO-fortilin^ cells (Fig. 1, *K* and ***L***; KO; *n* = 3, *p* = 0.004). These results further support a direct interaction between fortilin and MEF2C and show that this interaction occurs mainly in the nucleus (Fig. 1*K*, WT, merge). A subcellular fractionation assay of 293T cells revealed that MEF2C was predominantly localized in the nucleus, whereas fortilin not only was distributed mainly in the nucleus but was also present in the cytosol (24) (Fig. S1*H*).

These data from MST, *in vitro* co-IP Western blotting, *in vivo* forward and reverse co-IP Western blotting, and *in situ* PLA collectively suggest that fortilin and MEF2C specifically interact with each other and do so in the nucleus.

**Figure 2:**
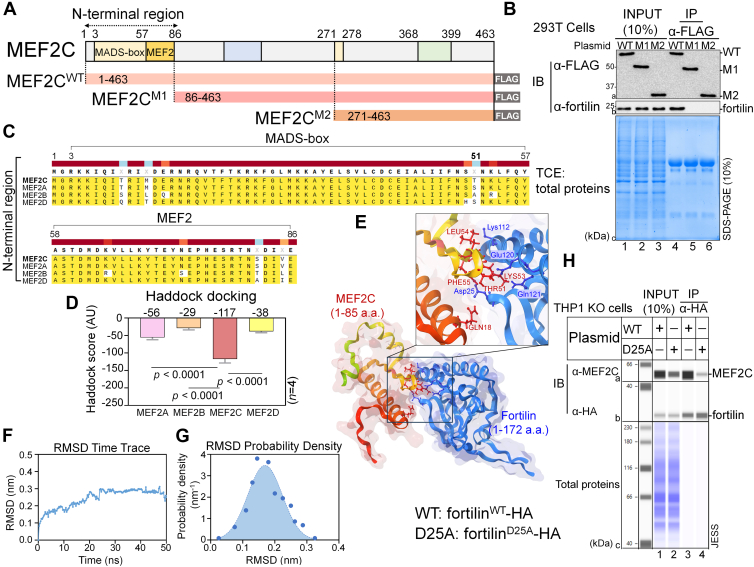
**Fortilin binds the N-terminal domain of MEF2C.***A,* MEF2C deletion mutants. Plasmids encoding MEF2C^WT^ and two deletion mutants, MEF2C^M1^ and MEF2C^M2^, lacking residues 1 to 85 and 1 to 270 of MEF2C, respectively, were generated on the pEZ mammalian expression vector. *B, in vivo* co-IP analysis of MEF2C deletion mutants. TCLs from 293T cells transfected with pEZ-MEF2C^WT^-FLAG, pEZ-MEF2C^M1^-FLAG, or pEZ-MEF2C^M2^-FLAG plasmid were subjected to IP in which FLAG-tagged MEF2C proteins were pulled down by α-FLAG Ab and the presence of coimmunoprecipitated fortilin was assessed by Western blotting using α-fortilin Ab. *C,* sequence alignment of the N-terminal regions of MEF2A, MEF2B, MEF2C, and MEF2D encompassing the MADS-box (residues 3–57) and MEF2 (residues 58–86) domains. Consensus amino acid residues among all four proteins are shown in *bold black*. Amino acids identical to those in MEF2C are highlighted in *yellow*. Threonine at position 51, which is uniquely present in MEF2C but absent in MEF2A, MEF2B, and MEF2D, is indicated in *bold type*. *D,* HADDOCK scores for fortilin binding to MEF2A, MEF2B, MEF2C, and MEF2D (*n* = 4 per the best cluster, one-way ANOVA with Tukey’s multiple comparisons). *E,* representative docking model showing full-length fortilin (*blue*) in complex with the N-terminal region of MEF2C *(red*). Ribbon rendering of the complex is shown with transparent surface representations for both proteins. Amino acid residues critically contributing to the binding interface are indicated for fortilin (Asp^25^, Lys^112^, Glu^120^, and Gln^121^) in *blue* and MEF2C (GLN^18^, THR^51^, LYS^53^, LEU^54^, and PHE^55^) in *red*. *F* and *G,* molecular dynamics simulations of the fortilin–MEF2C complex showing the RMSD time trace (*F*) and RMSD probability distribution (*G*). *H,* co-IP analysis of the fortilin^D25A^ mutant. THP1^KO-fortilin^ cells were transiently transfected with pCS mammalian expression vectors encoding HA-tagged WT fortilin (fortilin^WT^) or the mutant (fortilin^D25A^). TCLs were immunoprecipitated using an anti-HA antibody, and coimmunoprecipitated MEF2C was detected by Western blotting. AU, arbitrary unit; FLAG, an epitope-tag consisting of DYKDDDDK; fortilin^WT^-HA, pCS-fortilin^WT^-3×HA plasmid that expresses the WT MEF2C fused to the three hemagglutinin (HA) epitope tag repeats at its C-terminal end; fortilin^D25A^-HA, pCS-fortilin^D25A^-3×HA; IP, immunoprecipitation; M1, pEZ-MEF2C^M1^-FLAG (amino acids 86–463); M2, pEZ-MEF2C^M1^-FLAG (amino acids 271–463); MADS-box, MCM1-Agamous-Deficiens-Serum Response Factor box; MEF2, myocyte enhancer factor-2; MEF2C^WT^, WT MEF2C (1–463 amino acids); MEF2C^M1^, a deletion mutant of MEF2C lacking the N-terminal MADS-box and MEF2 domains and containing amino acids 86 to 463 of WT MEF2C; MEF2C^M2^, a deletion mutant containing amino acids 271 to 463 of WT MEF2C; α-MEF2C, anti-MEF2C antibody; α-HA, anti-HA-tag antibody; TCL, total cell lysate; THP1^WT-fortilin^, THP1 cells expressing WT fortilin; THP1^KO-fortilin^, THP1 cells in which the fortilin genes have been deleted by the CRISPR–Cas9 technology; WT, pEZ-MEF2C^WT^-FLAG plasmid that expresses the WT MEF2C (amino acids 1–463) fused to the FLAG epitope tag at its C-terminal region.

The 463 amino acid transcriptional factor MEF2C contains in its N-terminal region the 55 amino acid MADS-box (amino acids 3–57), which mediates homodimerization and binding to the target DNA sequences, and the 29 amino acid MEF2 domain (amino acids 58–86), which modulates DNA binding affinity and cofactor interactions (32, 33, 34) (Fig. 2*A*). We refer to the first 86 amino acids of MEF2C as the N-terminal region hereafter. The remaining 377 amino acid C-terminal region (amino acids 87–463), also known as the transactivation domain, contains the eight amino acid β transcription enhancer domain (amino acids 271–278) and the 32 amino acid transcription repressor domain (amino acids 368–399), which are required for transcriptional activation of MEF2C target genes (32) (Fig. 2*A*).

To test whether fortilin binds the N-terminal region of MEF2C that is involved in DNA binding (32, 33, 34) (as it does with the DNA-binding domain of p53 (11)), we generated complementary DNA (cDNA) constructs encoding two FLAG-tagged MEF2C deletion mutants—M1 (contains amino acids 86–463) and M2 (amino acids 271–463)—on the pEZ mammalian expression vector (pEZ-MEF2C^M1^-FLAG, pEZ-MEF2C^M2^-FLAG; control = pEZ-MEF2C^WT^-FLAG). We then transiently transfected 293T cells with these plasmids, generated TCLs (Fig. 2*B*, panels a1–a3), subjected them to co-IP Western blot analyses in which FLAG-tagged WT and mutant MEF2C proteins were immunoprecipitated by α-FLAB mAb (Fig. 2*B*, panels a4–a6), and then probed the precipitates for fortilin using α-fortilin mAb. We found that MEF2C^WT^ (Fig. 2*B*, b4), but not MEF2C^M1^ (Fig. 2*B*, b5) or MEF2C^M2^ (Fig. 2*B*, b6), was capable of interacting with fortilin, suggesting that fortilin binds the N-terminal region of MEF2C.

Using phylogenetic and homology analyses, we found that MEF2C and MEF2A are phylogenetically and structurally more similar to each other, whereas MEF2C shares less similarity with MEF2D or MEF2B (Fig. S2, *A* and *B*). In addition, the N-terminal region of MEF2C that interacts with fortilin (Fig. 2, *A* and *B*) is nearly identical to that of MEF2A, MEF2B, and MEF2D, except for the 51st residue: MEF2C contains Thr at that position, whereas MEF2A, MEF2B, and MEF2D contain serine (Ser), alanine (Ala), and Ser, respectively (Fig. 2*C*). This finding suggests that fortilin may interact with residues flanking this nonconserved amino acid (Thr^51^, Fig. 2*C*).

As we experimentally showed, fortilin binds the N-terminal region of MEF2C (Fig. 2*B*), which is highly conserved among all MEF2 family members (Fig. 2*C*). To better understand the structural basis of the fortilin–MEF2C interaction, we performed molecular docking simulations between fortilin and the N-terminal regions of MEF2A, MEF2B, MEF2C, and MEF2D using the High Ambiguity Driven protein–protein DOCKing (HADDOCK) 2.4 platform (35) and protein structures obtained from the Protein Data Bank (PDB). Consistent with the results of MST assays (Fig. 1, *B–E*) and co-IP assays (Figs. 1, *F–J* and S1*G*), the interaction between fortilin and MEF2C was the strongest among all MEF2 family members, as evidenced by the HADDOCK score for MEF2C being the lowest (most favorable) and significantly lower than those for MEF2A, MEF2B, and MEF2D (Fig. 2*D*; MEF2C *versus* MEF2A, MEF2B, and MEF2D = 117.23 ± 11.34 *versus* −55.60 ± 6.50, −28.90 ± 5.20, and −38.10 ± 4.10 arbitrary units [AU], respectively; *n* = 4, *p* < 0.001 for all comparisons), supporting the validity of the docking model. Further examination of the binding interface of the docking pose (Fig. 2*E*) revealed that Asp^25^, lysine 112 (Lys^112^), glutamic acid 120 (Glu^120^), and glutamine 121 (Gln^121^) of fortilin and GLN^18^, THR^51^, LYS^53^, leucine 54 (LEU^54^), and phenylalanine (PHE^55^) of MEF2C are located within 2 Å of the interface, suggesting that these amino acids critically contribute to the fortilin–MEF2C interaction (Fig. 2*E*). Notably, THR^51^ of MEF2C was identified as one of the five key residues involved in fortilin binding in the docking model (Fig. 2*E*) and is uniquely present in this position in MEF2C but not in MEF2A, MEF2B, or MEF2D (Fig. 2*C*), to which fortilin does not bind (Fig. 1, *B*, *C*, *E*, *F* and *G*, *I*).

To further validate the docking pose, we performed molecular dynamics simulations of the fortilin–MEF2C complex. We observed that, following an initial equilibration phase, the RMSD of the complex stabilized at approximately 0.3 nm after ∼20 ns and remained stable through 50 ns, with the interacting residues largely unaffected (Fig. 2*F*). The RMSD probability distribution exhibited a single peak centered at ∼0.18 nm with a narrow width, suggesting a stable, native-like conformational ensemble with minimal deviation of the reference structure (Fig. 2*G*). Finally, to experimentally validate the docking interface, we mutated Asp^25^ of fortilin, one of the four interface residues, to Ala (fortilin^D25A^) and evaluated its interaction with MEF2C by co-IP Western blotting (Fig. 2*H*). We transfected THP1^KO-fortilin^ cells with mammalian expression vectors encoding fortilin^WT^-hemagglutinin (HA) or fortilin^D25A^-HA, subjected the TCLs to IP using an anti-HA mAb, and assessed coimmunoprecipitated MEF2C. We detected comparable levels of immunoprecipitated fortilin-HA in both conditions (Fig. 2*H*, b3 *versus* b4); however, fortilin^WT^-HA coimmunoprecipitated markedly more MEF2C than did fortilin^D25A^ (Fig. 2*H*, a3 *versus* a4), suggesting that the D25A mutation of fortilin weakened fortilin–MEF2C binding. The results of MST analysis (Fig. 1, *B–E*), co-IP Western blot analysis of MEF2 proteins (Fig. 1, *F–J)*, and co-IP Western blot analysis of the fortilin^D25A^ mutant (Fig. 2*H*) are all consistent with the presented HADDOCK docking model (Fig. 2, *D–G*).

### Fortilin protects MEF2C against proteasome-mediated degradation

**Figure 3:**
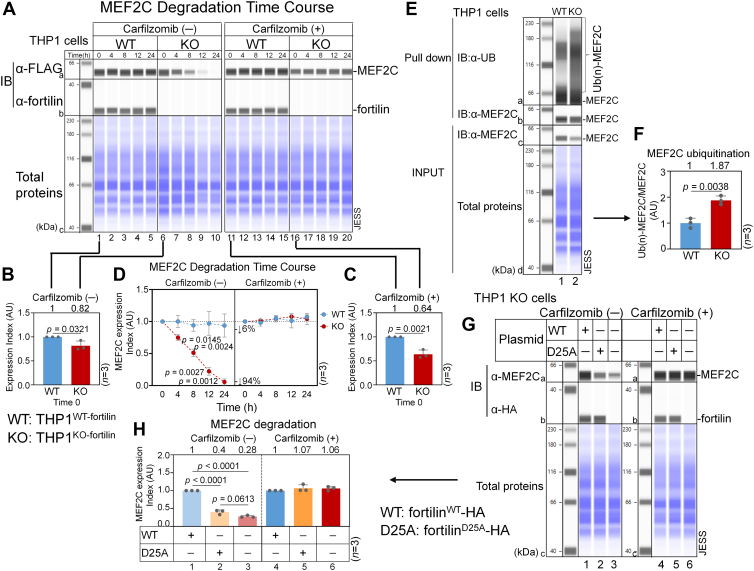
**Fortilin–MEF2C interaction mitigates the ubiquitination and proteasomal degradation of MEF2C.***A*–*D,* time-course analysis of MEF2C degradation in the presence or the absence of carfilzomib using the JESS capillary-based Western blot system. THP-1 cells were transiently transfected with pEZ-MEF2C^WT^-FLAG, incubated for 48 h, treated with CHX in the presence or the absence of carfilzomib (defined as time 0), and harvested at the indicated time points. TCLs were analyzed by IB with α-FLAG and α-fortilin Abs to assess MEF2C^WT^-FLAG and fortilin expression, respectively. Total protein loading was visualized using the JESS total protein detection module (*A*). Quantitative analysis of MEF2C expression and degradation in THP1^WT-fortilin^ and THP1^KO-fortilin^ cells treated with and without carfilzomib. Four experimental groups were analyzed—THP1^WT-fortilin^ ± carfilzomib and THP1^KO-fortilin^ ± carfilzomib. MEF2C expression indices (AU) were calculated at each time point using JESS Compass software by dividing the area under the MEF2C peak by the total protein signal within the same capillary (“in-capillary normalization”). Absolute MEF2C expression levels at time 0 were first compared between THP1^WT-fortilin^ and THP1^KO-fortilin^ cells in the absence (*B*) and presence (*D*) of carfilzomib. Data are presented as means ± standard deviation with *p* values indicated (*n* = 3, two-sample *t* test). MEF2C degradation kinetics were then assessed by normalizing MEF2C expression indices at each time point to the corresponding time 0 value within each group and comparing degradation profiles between THP1^WT-fortilin^ and THP1^KO-fortilin^ cells in the absence and presence of carfilzomib (*n* = 3, two-sample *t* test) (*C*). *E* and *F,* MEF2C ubiquitination assay. MEF2C was immunoprecipitated from the TCLs of THP1^WT-fortilin^ and THP1^KO-fortilin^ cells using an anti-MEF2C Ab, and ubiquitinated MEF2C was detected by IB with an anti-ubiquitin Ab (*E*). MEF2C ubiquitination indices were calculated by normalizing the total signal intensity of ubiquitinated MEF2C to the signal intensity of unmodified MEF2C in the TCLs (*n* = 3, two-sample *t* test) (*F*). *G* and *H,* effect of fortilin binding to MEF2C on MEF2C degradation. THP1^KO-fortilin^ cells were transfected with plasmids encoding fortilin^WT^-HA or fortilin^D25A^-HA (an MEF2C-binding–deficient mutant), treated with CHX, and incubated for 12 h in the presence or the absence of carfilzomib. TCLs were analyzed by Western blotting using anti-MEF2C and anti-HA Abs (*G*). MEF2C expression indices were calculated by normalizing the area under the curve of the MEF2C peak to total protein using Compass software (ProteinSimple, *n* = 3, ANOVA with Tukey’s multiple comparisons) (*H*). AU, arbitrary unit; carfilzomib, proteasome inhibitor; CHX, cycloheximide; α-FLAG, anti-FLAG antibody (Ab); α-fortilin, anti-fortilin Ab; fortilin^D25A^-HA, a HA-tagged MEF2C-binding–deficient fortilin mutant in which the 25th aspartic acid (*D*) is mutated to alanine (*A*); fortilin^WT^-HA, HA-tagged WT fortilin; IB, immunoblot; α-MEF2C, anti-MEF2C Ab; MG132, carbobenzoxy-L-leucyl-L-leucyl-L-leucine (proteasome inhibitor); TCL, total cell lysate; THP1^KO-fortilin^, THP1 cells in which the fortilin gene was deleted by CRISPR–Cas9 (KO); THP1^WT-fortilin^, THP1 cells expressing WT fortilin (WT); α-UB, anti-ubiquitin Ab; UB(n)-MEF2C, ubiquitinated MEF2C.

Fortilin binds to MCL1 (36), PRX1 (12), and CTNNA3 (9) and protects them against proteasome-mediated degradation. In addition, previous studies showed that MEF2C undergoes proteasome-mediated degradation (37, 38, 39). To test whether fortilin protects MEF2C from proteasome-mediated degradation, we transiently transfected THP1^WT-fortilin^ and THP1^KO-fortilin^ cells with the pEZ-MEF2C^WT^-FLAG mammalian-expression vector. After allowing MEF2C^WT^ to express for 48 h, we treated the cells with the protein synthesis inhibitor cycloheximide (CHX) (40) in the presence or the absence of the irreversible proteasome inhibitor, carfilzomib (41). CHX blocks translational elongation and thereby prevents *de novo* protein synthesis (40). We then harvested the cells at multiple time points (0, 4, 8, 12, and 24 h) and analyzed their lysates using the JESS system, which is an automated, capillary-based, highly quantitative Western blot platform (ProteinSimple) (42, 43) (Fig. 3*A*).

At time 0, defined as 48 h after transfection with the expression vector, MEF2C^WT^ expression levels were significantly lower in THP1^KO-fortilin^ cells than in THP1^WT-fortilin^ cells in both the carfilzomib (−) (Fig. 3*B*) and carfilzomib (+) (Fig. 3*C*) groups. When MEF2C^WT^ expression levels were normalized to 1.0 at time 0, the protein degraded more rapidly in THP1^KO-fortilin^ cells than in THP1^WT-fortilin^ cells in the absence of carfilzomib (Fig. 3*D*) (THP1^KO-fortilin^ *versus* THP1^WT-fortilin^ cells = 94% *versus* 6% at 24 h), and the MEF2C expression index was significantly lower in THP1^KO-fortilin^ cells than in THP1^WT-fortilin^ cells at times 4, 8, 12, and 24 h (Fig. 3*D*, carfilzomib(−)). In contrast, MEF2C^WT^ barely degraded in THP1^KO-fortilin^ and THP1^WT-fortilin^ cells in the presence of carfilzomib (Fig. 3*D*), with no significant differences in the MEF2C expression index between the two cell types at any time point (Fig. 3*D*, carfilzomib(+)).

To further confirm that these effects were attributable to proteasome inhibition, we repeated the experiment using another proteasome inhibitor, MG132 (44). We found that the proteasome inhibitor similarly abolished the differential degradation of MEF2C between THP1^WT-fortilin^ and THP1^KO-fortilin^ cells (Fig. S3, *A–D*). Taken together, these data suggest that fortilin protects MEF2C from proteasome-mediated degradation.

To test whether the lack of fortilin results in increased ubiquitination of MEF2C, we first treated THP1^WT-fortilin^ (WT) and THP1^KO-fortilin^ (KO) cells with carfilzomib and ML364, a deubiquitinase inhibitor (45), for 12 h. We then subjected the TCLs to IP by an anti-MEF2C Ab and assessed the degree of ubiquitination of immunoprecipitated MEF2C by Western blotting with an antiubiquitin Ab. Although THP1^KO-fortilin^ cells contained lower steady-state levels of MEF2C than did THP1^WT-fortilin^ cells (Fig. 3*E*, c1 *versus* c2) as expected, the anti-MEF2C Ab immunoprecipitated comparable amounts of MEF2C from both cell types (Fig. 3*E*, b1 *versus* b2). Under these conditions, THP1^KO-fortilin^ cells exhibited more extensive ubiquitination of MEF2C compared with THP1^WT-fortilin^ cells (Fig. 3*E*, a1 *versus* a2; Fig. 3*F*, *n* = 3, *p* = 0.0038), indicating that the lack of fortilin led to increased ubiquitination of MEF2C.

To evaluate whether direct interaction between fortilin and MEF2C is required to prevent proteasomal degradation of MEF2C, we transiently expressed either WT fortilin (fortilin^WT^) or an MEF2C-binding–deficient mutant (fortilin^D25A^) in THP1^KO-fortilin^ cells. We treated the cells with CHX and incubated them for 12 h in the presence or the absence of the proteasome inhibitor carfilzomib. We then subjected the TCLs to Western blot analyses using anti-fortilin and anti-MEF2C Abs to determine the expression levels of fortilin (fortilin^WT^ and fortilin^D25A^) and MEF2C. We found that fortilin^WT^ and fortilin^D25A^ were expressed at comparable levels in THP1^KO-fortilin^ cells (Fig. 3*G*, b1 *versus* b2). In contrast, MEF2C expression was markedly lower in fortilin^D25A^-expressing cells than in fortilin^WT^-expressing cells (Fig. 3*G*, a1 *versus* a2; Fig. 3*H*, columns 1 *versus* 2, *n* = 3, *p* < 0.0001) in the absence of carfilzomib. Moreover, MEF2C levels in fortilin^D25A^-expressing cells were comparable to those observed in mock-transfected THP1 cells (Fig. 3*G*, a2 *versus* a3; Fig. 3*H*, columns 2 *versus* 3, *n* = 3, *p* = 0.0613). Strikingly, these differences were completely abolished upon treatment with carfilzomib (Fig. 3*G*, a4, a5, and a6; Fig. 3*H*, columns 4, 5, and 6, *n* = 3). These data, when taken together, indicate that binding of fortilin to MEF2C is required to protect MEF2C from ubiquitination and subsequent proteasome-mediated degradation.

### Fortilin promotes MEF2C phosphorylation

The transcriptional activity of MEF2C is known to be enhanced by phosphorylation (39, 46, 47, 48, 49, 50, 51). Because fortilin increases the *MEF2C* transcript levels (Fig. 1*A*), we hypothesized that fortilin may enhance MEF2C phosphorylation. To test this premise, we first transiently transfected THP1^WT-fortilin^ (WT) and THP1^KO-fortilin^ (KO) cells with the pEZ-MEF2C^WT^-FLAG mammalian-expression plasmid vector, incubated them for 48 h, lysed them, and immunoprecipitated FLAG-tagged MEF2C^WT^ from the TCLs using anti-FLAG M2 magnetic beads. To avoid contamination from immunoglobulin heavy and light chains, we eluted immunoprecipitated MEF2C^WT^-FLAG using glycine under nondenaturing conditions. Next, we resolved the eluates by SDS-PAGE and stained them sequentially with Pro-Q Diamond (52) to detect phosphorylated MEF2C^WT^ and SYPRO Ruby (53) to visualize total immunoprecipitated MEF2C^WT^ (Fig. S4*A*).

**Figure 4:**
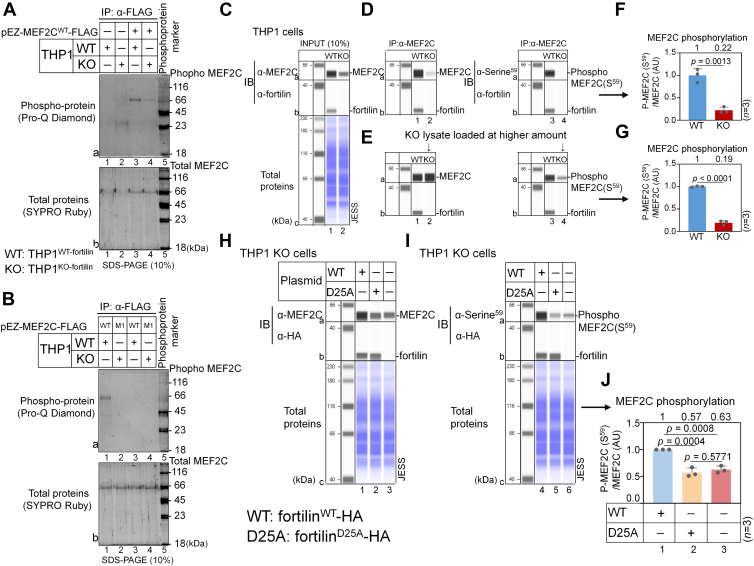
**Fortilin promotes the phosphorylation of MEF2C at Ser59 in the N-terminal region.***A,* phosphorylation status of WT MEF2C in THP1^WT-fortilin^ and THP1^KO-fortilin^ cells. THP1^WT-fortilin^ and THP1^KO-fortilin^ cells were transiently transfected with the pEZ-MEF2C^WT^-FLAG plasmid (mock transfection served as a control). MEF2C^WT^-FLAG was immunoprecipitated from the TCLs using an α-FLAG Ab and subjected to SDS-PAGE. Phosphorylated MEF2C^WT^ protein was visualized by Pro-Q Diamond staining (*top panel*), and total MEF2C^WT^ protein was visualized by SYPRO Ruby staining (*bottom panel*). *B,* comparison of the phosphorylation status of MEF2C^WT^ and MEF2C^M1^ in the presence (THP1^WT-fortilin^) and absence (THP1^KO-fortilin^) of fortilin. THP1^WT-fortilin^ and THP1^KO-fortilin^ cells were transiently transfected with either pEZ-MEF2C^WT^-FLAG or pEZ-MEF2C^M1^-FLAC plasmids. FLAG-tagged MEF2C^WT^ and MEF2C^KO^ proteins were then immunoprecipitated from the TCLs using an α-FLAG Ab and subjected to SDS-PAGE. Phosphorylated MEF2C proteins were visualized by Pro-Q Diamond staining (*top panel*), and total MEF2C proteins were visualized by SYPRO Ruby staining (*bottom panel*). *C*–*G,* evaluation of MEF2C phosphorylation at Ser^59^ in the presence and absence of fortilin. Endogenous MEF2C was immunoprecipitated from the TCLs of THP1^WT-fortilin^ (WT) and THP1^KO-fortilin^ (KO) cells using anti-MEF2C antibody. Total immunoprecipitated MEF2C and Ser^59^-phosphorylated MEF2C were analyzed using the JESS Western blot system (*C*–*E*). MEF2C phosphorylation indices were calculated from three independent experiments as the ratio of the area under the curve of phosphorpho-MEF2C signal to that of the total MEF2C signal and expressed in AU. A two-sample *t* test was used to compare the means of the WT and KO phosphorylation indices (*F* and *G*). *H*–*J,* evaluation of the role of fortilin–MEF2C binding in maintaining MEF2C phosphorylation at Ser^59^. THP1^KO-fortilin^ cells were transiently transfected with plasmids encoding 3xHA-tagged fortilin^WT^ or fortilin^D25A^ or with nothing. TCLs were analyzed using the JESS Western blot system with α-MEF2C, α-HA, and α-phospho-Ser^59^-MEF2C Abs (*H*, *I*). The MEF2C phosphorylation index was calculated as the ratio of Ser^59^-phosphorylated MEF2C to total MEF2C (*n* = 3; one-way ANOVA with Tukey’s multiple-comparisons test) (*J*). AU, arbitrary unit; α-FLAG, anti-FLAG antibody (Ab); fortilin^D25A^-HA, pCS-fortilin^D25A^-3×HA plasmid that expresses the MEF2C-binding–deficient fortilin^D25A^ mutant fused to the three hemagglutinin (HA) epitope tag repeats at its C-terminal end; fortilin^WT^-HA, pCS-fortilin^WT^-3×HA plasmid that expresses the WT fortilin fused to the three HA epitope tag repeats at its C-terminal end; α-HA, anti-HA Ab; IB, immunoblot; IP, immunoprecipitation; INPUT, 10% of TCLs used for IP; JESS, an automated capillary-based quantitative Western blot system; KO, THP1^KO-fortilin^; M1, pEZ-MEF2C^M1^-FLAG; α-MEF2C, anti-MEF2C Ab; pEZ-MEF2C^WT^-FLAG, a mammalian expression plasmid containing the construct for FLAG-tagged MEF2C; Pro-Q Diamond, staining for phosphorylated protein; α-Ser^59^, anti-MEF2C-Ser^59^ Ab; SYPRO Ruby, staining for total protein; TCL, total cell lysate; WT, THP1^WT-fortilin^.

MEF2C^WT^-FLAG was successfully immunoprecipitated from the TCL of pEZ-MEF2C^WT^-FLAG–transfected cells in comparable amounts across groups, as visualized by SYPRO-Ruby staining (Fig. 4*A*, b3, b4). Notably, phosphorylation of MEF2C^WT^-FLAG was observed only in the TCL from THP1^WT-fortilin^ cells (Fig. 4*A*, a3) and was absent in the TCL from THP1^KO-fortilin^ cells (Fig. 4*A*, a4). These data suggest that MEF2C is more heavily phosphorylated in the presence of fortilin in the cell and that fortilin promotes the phosphorylation of MEF2C.

As shown in the PhosphoSite Plus database (54), MEF2C phosphorylation occurs throughout the protein, including in the N-terminal region containing the highly conserved MADS-box and MEF2 domains, as well as in the C-terminal region, which is more divergent among MEF2 family members (Figs. 2*C* and S2, *A* and *B*). Because fortilin promotes MEF2C phosphorylation (Fig. 4*A*) and binds to its N-terminal region (Fig. 2*B*), we hypothesized that fortilin facilitates phosphorylation specifically within the N-terminal region of MEF2C. To test this premise, we first transiently transfected THP1^WT-fortilin^ and THP1^KO-fortilin^ cells with either pEZ-MEF2C^WT^-FLAG or pEZ-MEF2C^M1^-FLAG. pEZ-MEF2C^M1^-FLAG lacks the N-terminal region (amino acids 1–85) of MEF2C (Fig. 2*A*) and does not bind fortilin (Fig. 2*B*). We then immunoprecipitated MEF2C^WT^-FLAG and MEF2C^M1^-FLAG using anti-FLAG M2 magnetic beads, eluted immunoprecipitated MEF2C^WT^-FLAG and MEF2C^M1^-FLAG under nondenaturing conditions, resolved the eluates by SDS-PAGE, and sequentially stained them first with Pro-Q Diamond (52) to detect phosphorylated MEF2C proteins and then with SYPRO Ruby (53) to visualize total MEF2C proteins (Fig. S4*A*). Both MEF2C^WT^-FLAG (WT) and MEF2C^M1^-FLAG (M1) were successfully immunoprecipitated in comparable amounts from THP1^WT-fortilin^ and THP1^KO-fortilin^ cells, as visualized by SYPRO Ruby staining (53) (Fig. 4*B*, b1–4). Pro-Q Diamond staining revealed a phospho-protein band corresponding to MEF2C^WT^-FLAG (Fig. 4*B*, a1) in THP1^WT-fortilin^ cells, but a band for MEF2C^M1^-FLAG was not detected (Fig. 4*B*, a2). In contrast, no phospho-specific signal was detected for either MEF2C^WT^-FLAG (Fig. 4*B*, a3) or MEF2C^M1^-FLAG (Fig. 4*B*, a4) in THP1^KO-fortilin^ cells. These findings suggest that fortilin-dependent phosphorylation of MEF2C occurs predominantly in the N-terminal region (amino acids 1–85).

Within the N-terminal region of MEF2C, four potential phosphorylation sites have been reported (54): Thr^20^ (46), Ser^59^ (39, 47), Thr^80^ (48), and Ser^82^ (54). Among them, phosphorylation of Ser^59^ has been most rigorously associated with the functional activation of MEF2C as a transcriptional factor (39, 47). Notably, Molkentin *et al.* (47) demonstrated that phosphorylation at Ser^59^ leads to a fivefold increase in DNA binding and transcriptional activities of MEF2C. To test whether fortilin promotes MEF2C phosphorylation at Ser^59^, we immunoprecipitated endogenous MEF2C from THP1^WT-fortilin^ and THP1^KO-fortilin^ cells and immunoblotted the immunoprecipitated MEF2C with an anti-phospho-Ser^59^-MEF2C (α-p-MEF2C) Ab. We calculated an MEF2C phosphorylation index as the ratio of Ser^59^-phosphorylated MEF2C (p-MEF2C) to total immunoprecipitated MEF2C using α-p-MEF2C and α-total-MEF2C antibodies on the JESS platform. Consistent with the results shown in Figure 3, JESS analysis showed that THP1^KO-fortilin^ cells expressed lower total MEF2C levels compared with THP1^WT-fortilin^ cells (Fig. 4*C*, a1 *versus* a2), which resulted in reduced recovery of immunoprecipitated MEF2C (Fig. 4*D*, a1 *versus* a2). Under these conditions, THP1^KO-fortilin^ cells exhibited a fourfold lower MEF2C phosphorylation index than THP1^WT-fortilin^ cells (Fig. 4*D*, a3 *versus* a4; Fig. 4*F*, *n* = 3, *p* = 0.0013). When we analyzed comparable amounts of immunoprecipitated MEF2C (Fig. 4*E*, a1 *versus* a2), THP1^KO-fortilin^ cells showed a fivefold reduction in the MEF2C phosphorylation index relative to THP1^WT-fortilin^ cells (Fig. 4*E*, a3 *versus* a4; Fig. 4*G*, *n* = 3, *p* < 0.0001). Finally, when we directly probed the TCLs from THP1^WT-fortilin^ and THP1^KO-fortilin^ cells with α-total-MEF2C and α-p-MEF2C (Fig. S4*B*), we again observed a markedly lower MEF2C phosphorylation index in THP1^KO-fortilin^ cells (approximately 11-fold lower than in THP1^WT-fortilin^ cells) (Fig. S4*C*, *n* = 3, *p* < 0.0001). These data, when taken together, suggest that fortilin promotes MEF2C phosphorylation at Ser^59^.

To evaluate whether fortilin–MEF2C binding is required to maintain MEF2C phosphorylation at Ser^59^, which is the only site within the N-terminal region of MEF2C and has been shown to activate its transcriptional activity (47), we transiently transfected THP1^KO-fortilin^ cells with plasmids encoding fortilin^WT^-HA or fortilin^D25A^-HA or nothing, incubated the cells in the presence or the absence of carlifzomib, and analyzed the TCLs using the JESS Western blot system with anti-MEF2C Ab and anti-phospho-Ser^59^-MEF2C Ab (Fig. 4, *H* and *I*). We calculated the MEF2C phosphorylation index as the ratio of Ser^59^-phosphorylated MEF2C to total MEF2C. We found that the phosphorylation indices were approximately 40% lower in cells transfected with fortilin^D25A^ or untransfected THP1^KO-fortilin^ cells compared with cells transfected with fortilin^WT^ plasmid (Fig. 4*J*; *n* = 3, *p* = 0.0004 for fortilin^WT^ *versus* fortilin^D25A^, *p* = 0.0008 for fortilin^WT^ *versus* no transfection). These data suggest that direct fortilin–MEF2C binding is required to maintain Ser^59^ phosphorylation of MEF2C, which is a modification that is critical for MEF2C transcriptional activity (47).

### Fortilin enhances the transcriptional activation of CTNNA3 by MEF2C

We have thus far demonstrated that fortilin directly binds to MEF2C (Fig. 1, *B–L*), engages MEF2C through its N-terminal region (Fig. 2*B*), protects MEF2C from ubiquitin–proteasome-mediated degradation (Figs. 3, *A–F* and S3) in a binding-dependent manner (Fig. 3, *G* and *H*), and promotes MEF2C phosphorylation within the N-terminal region (amino acids 1–85), including at Ser^59^ (Fig. 4, *A–G*). Fortilin–MEF2C binding is required for MEF2C phosphorylation at Ser^59^ (Fig. 4, *H–J*), and phosphorylation of MEF2C at Ser^59^ has been shown to markedly enhance its DNA binding and transcriptional activity (39, 47).

**Figure 5:**
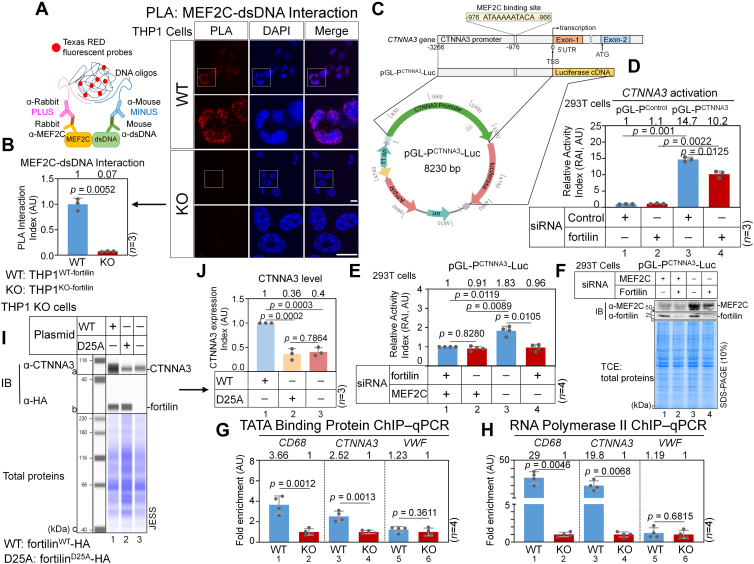
**Fortilin enhances MEF2C-mediated transcriptional activation of the *CTNNA3* promoter.***A* and *B,* PLA. Interaction between MEF2C and dsDNA evaluated by PLA. PLA was performed using rabbit α-MEF2C and mouse α-dsDNA Abs in THP1^WT-fortilin^ (WT, *top panels*) and THP1^KO-fortilin^ (KO, *bottom panels*) cells, with nuclei counterstained using DAPI (*blue*). *Red puncta* indicate that MEF2C and dsDNA are located within 30 nm of each other, signifying a specific interaction. The scale bar represents 10 μm. *White squares* indicate magnified regions shown below (*A*). The PLA interaction index was calculated as the number of PLA puncta per nucleus within a field of view and is expressed in arbitrary units (AU). Three fields were quantified for each sample, and three independent experiments were performed. Statistical significance was evaluated using a Welch’s two-sample *t* test (*B*). *C,* structure of the human *CTNNA3* gene (*top panel*) and pGL-P^CTNNA3^-Luc reporter vector (*bottom panels*). A high-probability MEF2C binding site was identified within the 3266-nucleotide *CTNNA3* promoter region using the JASPAR platform. This 3266-nucleotide *CTNNA3* promoter region that contains a top-scoring, predicted MEF2C binding motif (ATAAAAATACA) located from −976 to −966 relative to the transcription start site (TSS) was cloned upstream of the firefly luciferase reporter gene in the pGL vector to generate pGL-P^CTNNA3-Luc^ vector (total size = 8230 base pairs). *D,* dual luciferase reporter assay to assess the impact of fortilin on *CTNNA3* promoter activation. 293T cells were sequentially transfected with siRNA targeting fortilin (siRNA^fortilin^) or control siRNA (siRNA^control^), followed by cotransfection with pRL-null vector (*Renilla* luciferase, normalization control) and either pGL-P^Control-Luc^ or pGL-P^CTNNA3^-Luc reporter plasmids. Luciferase activity was measured 24 h post-transfection, and the RAI was calculated as the ratio of firefly to Renilla luciferase activity. Data are expressed as means ± standard deviation (*n* = 3 biological replicates) and were analyzed using two-way ANOVA followed by Tukey–Kramer multiple comparisons. *E* and *F,* dual luciferase reporter assay to determine whether MEF2C is required for fortilin-mediated activation of the *CTNNA3* promoter. 293T cells were transfected with one of the following siRNA combinations: (i) siRNA^MEF2C^ + siRNA^control^, (ii) siRNA^MEF2C^ + siRNA^fortilin^, (iii) siRNA^control^, or (iv) siRNA^fortilin^. Luciferase activity was measured 24 h post-transfection, and the RAI was calculated. Data are presented as means ± standard deviation (*n* = 4) and were analyzed using two-way ANOVA and Tukey’s multiple comparisons (*E*). Subsequently, cell lysates were subjected to Western blotting using α-MEF2C and α-fortilin Abs to confirm siRNA-mediated knockdown of target genes (*F*). *G* and *H,* TATA-binding protein (TBP) and RNA polymerase II (Pol II) chromatin immunoprecipitation (ChIP) of CTNNA3. ChIP assays were performed in THP1^WT-fortilin^ and THP1^KO-fortilin^ cells by crosslinking chromatin with formaldehyde, followed by immunoprecipitation of chromatin from the TCLs using an anti-TBP (*G*) or anti-Pol II (*H*) antibody coupled to Protein A/G beads. Normal IgG was used as a negative control. DNA from the immunoprecipitated chromatin was purified and analyzed by quantitative PCR (qPCR) using primers flanking the transcription start sites (TSSs) of *CTNNA3*, *CD68* (positive control), and *VWF* (negative control). TBP (*G*) and Pol II (*H*) occupancy indices for CTNNA3, CD68, and vWF were expressed as fold enrichment values (AU) relative to IgG (*n* = 4; Student's *t* test for TBP (*G*) and Welch’s *t* tests for Pol II (*H*). *I* and *J,* CTNNA3 expression in THP1^KO-fortilin^ cells overexpressing WT (fortilin^WT^) and an MEF2C-binding–deficient (fortilin^D25A^) fortilin. THP1^KO-fortilin^ cells were transiently transfected with plasmids encoding HA-tagged fortilin^WT^ or fortilin^D25A^. Untransfected THP1^KO-fortilin^ cells were used as controls. TCLs were analyzed using the JESS automated Western blot system with anti-HA and anti-CTNNA3 antibodies (*I*). The CTNNA3 expression index was calculated by normalizing the area under the CTNNA3 peak to the total protein signal and expressed as arbitrary units (AU) (*n* = 3; one-way ANOVA with Tukey’s multiple comparisons) (*J*). AU, arbitrary unit; α-CTNNA3, anti-CTNNA3 antibody; ChIP–qPCR, chromatin immunoprecipitation–quantitative PCR; DAPI, 4′,6-diamidino-2-phenylindole; α-dsDNA, anti–double-stranded DNA antibody (Ab); α-fortilin, anti-fortilin Ab; Fortilin WT, THP1^WT-fortilin^, THP1 cells expressing WT fortilin; Fortilin KO, THP1^KO-fortilin^, THP1 cells in which the fortilin genes have been deleted by the CRISPR–Cas9 technology; α-HA, anti-HA-epitope-tag antibody; IB, immunoblot; α-MEF2C, anti-MEF2C Ab; pGL-P^Control^, pGL-luciferase vector with no promoter; pGL-P^CTNNA3^-Luc, pGL luciferase vector in which the *CTNNA3* promoter is fused to the firefly luciferase construct; PLA, proximity ligation assay; Plasmid-D25A, pCS-fortilin^D25A^-3×HA plasmid that expresses the MEF2C-binding–deficient fortilin^D25A^ mutant fused to the three HA epitope tag repeats at its C-terminal end; Plasmid-WT, pCS-fortilin^WT^-3×HA plasmid that expresses the WT fortilin fused to the three hemagglutinin (HA) epitope tag repeats at its C-terminal end; RAI, relative activity index; TCE, 2,2,2-trichloroethanol; TCL, total cell lysate.

To test the hypothesis that fortilin promotes the binding of MEF2C to DNA *in vivo*, we conducted *in situ* PLA in THP1^WT-fortilin^ and THP1^KO-fortilin^ cells using mouse α-dsDNA and rabbit polyclonal α-MEF2C antibodies. The PLA revealed numerous red dots within the 4′,6-diamidino-2-phenylindole–stained nuclei of THP1^WT-fortilin^ cells (Fig. 5*A*, WT), whereas we did not observe any red dots in THP1^KO-fortilin^ cells (Fig. 5*A*, KO). We calculated the PLA interaction index as the number of PLA puncta per nucleus within a region of interest and expressed it in AU. The PLA interaction index for the MEF2C–dsDNA interaction was significantly greater in WT than in KO cells (Fig. 5*B*, *n* = 3, *p* = 0.0052). These results suggest that fortilin is required for MEF2C to interact with nuclear dsDNA.

Vanpoucke *et al.* (26) performed luciferase reporter assays in P19 embryonal carcinoma cells transiently cotransfected with the pEF6-MEF2C mammalian expression vector and a pGL3-luciferase reporter vector containing a human *CTNNA3* promoter construct (spanning positions from −3266 to 0 [the transcription start site (TSS)]; pGL3-P^CTNNA3^-Luc) (Fig. 5*C*) and found that MEF2C dose-dependently activated the *CTNNA3* promoter. To identify potential MEF2C binding sites within the *CTNNA3* promoter sequence, we utilized the MEF2C motifs (MA0497.1 and MA0497.2) from the latest JASPAR platform database (55) and conducted a scan on the platform using the sequence spanning from −3000 to 0. The scan identified just one high-confidence MEF2C binding DNA sequence, ATAAAAATACA (positions from −976 to −966, relative score = 91.6%) (Fig. 5*C*), which suggests that the *CTNNA3* promoter contains at least one MEF2C binding site in its proximal portion, likely driving MEF2C-induced *CTNNA3* transcription.

Next, to assess the role of fortilin in promoter-dependent transcriptional activation of the *CTNNA3* gene, we transfected 293T cells with either fortilin-silencing siRNA (siRNA^fortilin^) or control siRNA (siRNA^control^). We then conducted dual-luciferase reporter assays in which cells were cotransfected with pGL-P^CTNNA3^-Luc (Fig. 5*C*) and the pRL-Renilla luciferase vector. The reporter activation index was calculated as the ratio of firefly to Renilla luciferase activity. We found that the *CTNNA3* promoter was active under the baseline conditions in 293T cells and that fortilin knockdown significantly reduced promoter activity (Fig. 5*D*, columns 3 *versus* 4, *n* = 3, *p* = 0.0125), indicating that fortilin facilitates activation of the *CTNNA3* promoter.

Although we found that fortilin enhances the binding of MEF2C to DNA (Fig. 5, *A* and *B*) and is required for full activation of the *CTNNA3* promoter (Fig. 5*D*) and that MEF2C binds (Fig. 5*C*) and activates the *CTNNA3* promoter (26), the role of fortilin in MEF2C-mediated *CTNNA3* promoter activation remained unclear. We hypothesized that fortilin acts as a transcriptional cofactor of MEF2C and is required for MEF2C-dependent activation of the *CTNNA3* promoter. To test this, we conducted dual-luciferase reporter assays in four groups of 293T cells transfected with (a) either siRNA^control^ or siRNA^MEF2C^ and (b) either siRNA^control^ or siRNA^fortilin^. In the presence of MEF2C, fortilin knockdown significantly reduced the reporter activation index by 47.5% (Fig. 5*E*, columns 3 *versus* 4; *n* = 3, *p* = 0.0105), confirming that fortilin promotes *CTNNA3* promoter activity (as shown in Fig. 5*D*). Notably, however, fortilin knockdown had no additional effect in the absence of MEF2C: RAIs in the siRNA^MEF2C^siRNA^control^ and siRNA^MEF2C^siRNA^fortilin^ groups were similarly reduced and significantly lower than those in control cells (Fig. 5*E*, column 1 = 2 < 3, *n* = 3, *p* = 0.0119 for columns 1 *versus* 3). These findings suggest that fortilin cannot activate the *CTNNA3* promoter independently of MEF2C and that it instead potentiates MEF2C-mediated activation of the *CTNNA3* promoter. Western blot analyses confirmed successful knockdown of MEF2C and fortilin in their respective siRNA-treated groups (Fig. 5*F*). These data suggest that fortilin functions as a transcriptional cofactor of MEF2C.

To further validate the luciferase assay findings (Fig. 5, *C–F*) showing that fortilin promotes *CTNNA3* gene transcription, we performed a chromatin immunoprecipitation (ChIP)–qPCR assay to assess the recruitment and engagement of TATA-binding protein (TBP) and RNA polymerase II (hereafter, Pol II) at the *CTNNA3* TSS, using *CD68* and von Willebrand factor (*VWF*) as positive and negative controls, respectively (Fig. S5*A* and S5*B*). *CD68* is primarily transcribed in monocyte–macrophage lineage cells (56), and its expression is markedly reduced in the absence of fortilin (28). In contrast, *VWF* is robustly transcribed in endothelial cells but is minimally expressed in monocyte–macrophage cells, including THP1 cells (57). For TBP ChIP–qPCR, we treated THP1^WT-fortilin^ and THP1^KO-fortilin^ cells with 1% formaldehyde to crosslink protein–DNA complexes, generated TCLs, immunoprecipitated TBP using an anti-TBP Ab or IgG (negative control) coupled to Protein A/G beads, and analyzed the recovered DNA by qPCR using primer sets flanking the TSSs of *CTNNA3*, *CD68*, and *VWF*. We calculated TBP occupancy indices for CTNNA3 by normalizing the amount of *CTNNA3* TSS DNA immunoprecipitated with the anti-TBP Ab to that immunoprecipitated with rabbit normal IgG and expressed the results as AU. We calculated TBP occupancy indices for *CD68* and *VWF* in the same manner. For Pol II ChIP–qPCR, an anti-Pol II Ab was used in place of the anti-TBP Ab.

Consistent with prior reports (28), THP1^WT-fortilin^ cells exhibited greater TBP and Pol II occupancy at the *CD68* locus than did THP1^KO-fortilin^ cells (TBP, Fig. 5*G*, columns 1 *versus* 2, *n* = 4, *p* = 0.0012; Pol II, Fig. 5*H*, columns 1 *versus* 2, *n* = 4, *p* = 0.0046). In addition, TBP and Pol II occupancy indices for *VWF* were similar between THP1^WT-fortilin^ and THP1^KO-fortilin^ cells (TBP, Fig. 5*G*, columns 5 *versus* 6, *n* = 4, *p* = 0.3611; Pol II, Fig. 5*H*, columns 5 *versus* 6, *n* = 4, *p* = 0.6815). Under these conditions, THP1^WT-fortilin^ cells displayed significantly greater TBP and Pol II occupancy at the *CTNNA3* locus than THP1^KO-fortilin^ cells (TBP, Fig. 5*G*, columns 3 *versus* 4, *n* = 4, *p* = 0.0013; Pol II, Fig. 5*H*, columns 3 *versus* 4, *n* = 4, *p* = 0.0068). Together, these results suggest that fortilin promotes *CTNNA3* transcription and are consistent with the data from luciferase assays (Fig. 5, *C–F*).

Finally, to determine how the fortilin–MEF2C interaction impacts CTNNA3 protein expression, we transiently transfected THP1^KO-fortilin^ cells with (a) a fortilin^WT^-expressing plasmid, (b) a fortilin^D25A^-expressing plasmid, or (c) no plasmid and analyzed the TCLs using the JESS quantitative Western blot system. Fortilin expression levels were comparable between THP1^KO-fortilin^ cells overexpressing fortilin^WT^ and fortilin^D25A^ (Fig. 5*H*, b1 *versus* b2). Under these conditions, CTNNA3 expression was markedly lower in fortilin^D25A^-overexpressing cells than in fortilin^WT^-overexpressing cells (Fig. 5*H*, a1 *versus* a2; Fig. 5*I*, columns 1 *versus* 2; *n* = 3, *p* = 0.002). CTNNA3 expression was similar between fortilin^D25A^-overexpressing cells and cells lacking fortilin expression (Fig. 5*H*, a2 *versus* a3; Fig. 5*I*, columns 2 *versus* 3; *n* = 3, *p* = 0.7864). These data suggest that the fortilin–MEF2C interaction is required for normal CTNNA3 expression.

Prior research showed that MEF2C phosphorylation at Ser^59^ enhances its DNA-binding and transcriptional activity (47), and herein, we showed that MEF2C directly drives *CTNNA3* transcription (Fig. 5*E*). Together, these findings suggest that MEF2C phosphorylation plays an important role in driving CTNNA3 transcription. We also demonstrated that the fortilin–MEF2C interaction is required to (a) maintain MEF2C phosphorylation at Ser^59^ (Fig. 4, *H–J*), (b) protect MEF2C from proteasome degradation (Fig. 3, *G* and *H*), and (c) sustain CTNNA3 protein expression (Fig. 5, *H* and *I*). Collectively, these results support the premise that fortilin promotes CTNNA3 protein expression by stabilizing and activating MEF2C through direct binding.

**Figure 6:**
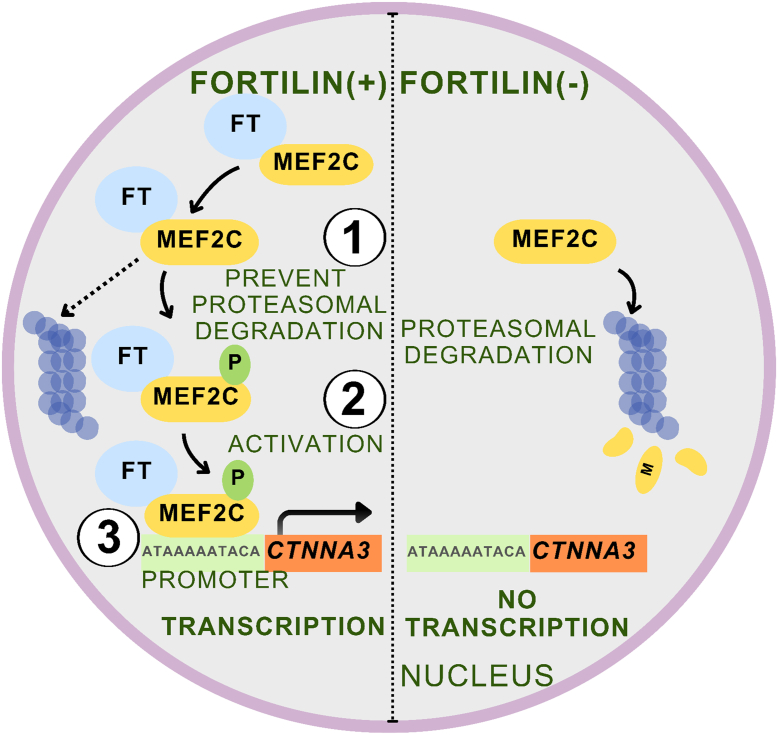
**Fortilin binds the transcription factor MEF2C, protects it against proteasomal degradation, enhances its phosphorylation, and transcriptionally activates the *CTNNA3* promoter.** Under normal conditions (Fortilin [+], *left panel*), fortilin binds MEF2C and protects it from proteasomal degradation **①**. In addition, fortilin facilitates the phosphorylation of MEF2C **②**, thereby enhancing its DNA binding capacity and promoting transcriptional activation of the *CTNNA3* gene **③**. In contrast, under fortilin-deficient conditions (Fortilin [–], *right panel*), MEF2C undergoes increased proteasomal degradation and becomes less phosphorylated at Ser^59^, which impairs its ability to bind the *CTNNA3* promoter and transcriptionally activate the gene, ultimately leading to cellular dysfunction. FT, fortilin; P, phosphorylation.

In conclusion, under normal conditions (Fig. 6, Fortilin [+]), fortilin binds to MEF2C, protects it against proteasomal degradation (Fig. 6, Fortilin [+] [1]), enhances its transcriptional activity by promoting phosphorylation (Fig. 6, Fortilin [+] [2]), and facilitates *CTNNA3* promoter activation by acting as a transcriptional cofactor of MEF2C (Fig. 6, Fortilin [+] [3]). Thus, fortilin is required for optimal MEF2C-mediated *CTNNA3* transcription. When cellular fortilin levels are reduced (Fig. 6, Fortilin [–]), MEF2C levels also decrease because of increased proteasomal degradation. The remaining MEF2C is less phosphorylated and, in the absence of fortilin as a cofactor, binds less effectively to its target DNA sequence, resulting in diminished activation of the *CTNNA3* promoter.

## Discussion

We conducted this study to elucidate how the loss of fortilin leads to decreased *CTNNA3* transcript levels in cells (Fig. 1*A*). We found that fortilin functions as a transcriptional cofactor (Fig. 5*E*), stabilizer (Fig. 3), and positive regulator of phosphorylation (Fig. 4) for MEF2C, which in turn binds to (Fig. 5, *A* and *B*) and activates (Fig. 5, *C–F*) the *CTNNA3* promoter and *CTNNA3* transcription (Fig. 5*G*) in a fortilin–MEF2C interaction–dependent fashion (Fig. 3, *G* and *H*, 4, *H–J*, and 5, *H* and *I*). These findings reveal a previously unreported mechanism by which fortilin regulates MEF2C and CTNNA3 (Fig. 6).

MEF2C differs from other MEF2 family proteins by being expressed very early in development, particularly in the heart (58) and brain (59), and it is essential for embryonic survival. Constitutive deficiency of MEF2C results in early embryonic lethality because of severe cardiac malformations (58). In contrast, other MEF2 family proteins are not essential for embryonic survival. Mice globally deficient in MEF2A survive until birth, although most die suddenly in the early postnatal period and exhibit right ventricular dilatation (60). Mice globally deficient in MEF2B are viable, fertile, and grossly normal except for exhibiting abnormal snout morphology (International Mouse Phenotype Consortium (61), https://www.mousephenotype.org/data/genes/MGI:104526). Mice globally deficient in MEF2D are also viable and fertile with only mild phenotypes (62, 63). Collectively, these findings suggest that the fortilin–MEF2C interaction likely plays a unique and critical role during development.

The finding that fortilin binds to MEF2C and protects it from proteasome-mediated degradation is not surprising because fortilin also binds PRX1 (12), MCL1 (36), and CTNNA3 (9) and protects them against proteasome-mediated degradation. Intriguingly, all the proteins that fortilin binds and protects against degradation promote cell survival (9, 12, 36), suggesting that antiapoptotic activity of fortilin may, at least partially, derive from its ability to stabilize prosurvival protein partners. What was unexpected is that fortilin positively regulates CTNNA3 at both the protein and transcriptional levels. As we previously reported, fortilin binds to CTNNA3 and protects it against phosphorylation and phosphorylation-dependent, proteasome-mediated degradation (9). In the current study, we found that fortilin also binds to MEF2C, a transcriptional factor that promotes *CTNNA3* transcription, and safeguards it from proteasome-mediated degradation. These findings raise the possibility that fortilin may regulate other proteins at both transcriptional and post-translational levels through similar mechanisms.

Fortilin can either facilitate or suppress the phosphorylation of its protein partners. For example, fortilin interacts with the antioxidant enzyme PRX1 and maintains it in a phosphorylated and functionally active state so that it can defend cells against reactive oxygen species–induced damage (12). In the present study, we showed that fortilin binds to MEF2C and potentiates its DNA-binding activity at the *CTNNA3* promoter by facilitating its phosphorylation (Fig. 4). In contrast, fortilin binds to the endoplasmic reticulum stress protein inositol-requiring enzyme type 1 and sustains it in an unphosphorylated state, preventing it from activating the apoptosis pathway (13). Similarly, fortilin interacts with the cardiac structural and survival protein CTNNA3, protecting it against phosphorylation and phosphorylation-dependent degradation (9). The mechanism by which fortilin determines whether to preserve its protein partners in a phosphorylated or an unphosphorylated state remains unknown and warrants further investigation. Finally, although four phosphorylation sites have previously been reported within the N-terminal region of MEF2C (54) (Thr^20^ (46), Ser^59^ (39, 47), Thr^80^ (48), and Ser^82^ (54)), MEF2C can also be phosphorylated in additional sites, including Ser^98^ (38), Ser^110^ (38), Ser^222^ (49), Thr^293^ (51), Thr^300^ (51), and Ser^387^ (51). Phosphorylation of these sites has been implicated in various biological processes and diseases, such as Ser^98^ and Ser^110^ in proteasome-mediated degradation (38), Ser^222^ in acute myeloid leukemia chemoresistance (49), and Thr^293^, Thr^300^, and Ser^387^ in c-Jun–mediated inflammation (51). For some of these phosphorylation sites, the responsible kinases have been identified, including mitogen-activated protein kinase p38 for Thr^293^, Thr^300^, and Ser^387^ (51) and Erk5 mitogen-activating kinase for Ser^59^ (39). In the current work, we focused on Ser^59^ because it has been most rigorously associated with the functional activation of MEF2C as a transcriptional factor (39, 47) and is located in the region of MEF2C to which fortilin binds. Nevertheless, it is possible that fortilin also modulates the phosphorylation of other MEF2C phosphorylation sites, and this possibility requires further investigation.

In addition to its localization in the cytosol (4) and extracellular space (5, 6, 7), fortilin is also present in the nucleus (4), where it is known to interact with transcriptional factors, such as p53 (64) and transcription factor 4 (TCF-4) (20). On one hand, fortilin binds to the sequence-specific DNA-binding domain of p53 and prevents it from engaging the p53-responsive element of *BAX*, thereby mitigating *BAX* gene expression (64). On the other hand, fortilin interacts with the N-terminal region of TCF-4 but not with its DNA-binding HMG box domain (20). Taken together with our current finding that fortilin binds the DNA-binding region of MEF2C (Fig. 2, *A* and *B*) and promotes MEF2C-mediated activation of the *CTNNA3* promoter (Fig. 5, *C–F*), these observations suggest that fortilin may function as either a positive (*e.g.*, MEF2C, TCF-4) or a negative (*e.g.*, p53) transcriptional regulator by either directly binding to the DNA-binding domain of transcriptional factors (*e.g.*, MEF2C, p53) or modulating their DNA-binding capacity through interactions at non–DNA-binding regions (TCF-4).

While the current study demonstrated that fortilin is an MEF2C-interacting protein, MEF2C has previously been reported to interact with several classes of proteins, including histone deacetylases (65, 66), transcriptional cofactors (67, 68), and transcriptional factors (69), which regulate its transcriptional activity, either positively (67, 68, 69) or negatively (65, 66). For example, human HDAC4 binds to the N-terminal region of MEF2C and prevents it from activating the c-jun promoter (65). Similarly, HDAC5 binds to MEF2C and prevents it from activating the *MEF2*-responsive element (66). EP300, a histone acetyltransferase and transcriptional cofactor, binds to the MADS-Box domain of MEF2C and enhances its transcriptional activities (67). TEF-1, a transcriptional cofactor, also interacts with the MADS-box domain of MEF2C and promotes its activation of the β-myosin heavy chain promoter (68). Sp1, a transcriptional factor, binds to the N-terminal region of MEF2C and enhances its activation of the *N*-methyl-d-aspartate receptor subunit 1 promoter (69). These interactions highlight the complexity of MEF2C regulation.

In this study, we mainly used human embryonic kidney 293T (HEK293T) cells and fortilin-deficient and fortilin-competent THP1 cells (THP1^KO-fortilin^ and THP1^WT-fortilin^ cells, respectively). We used HEK293T cells for biochemical (Figs. 1, *F–J*, 2*B*, and S1*H*) and transcriptional (Fig. 5, *D–F*) assays because of their high transfection efficiency and robust protein expression. We used THP1 cells because of the availability of THP1^KO-fortilin^ generated using CRISPR–Cas9 technology (9, 28), which provided a clean genetic background and allowed unambiguous interpretation of data as we assessed the role of fortilin in transcription of *CTNNA3* and other genes (Figs. 1*A*, and S1, *A–F*), the MEF2C–fortilin interaction by PLA (Fig. 1, *K and L*), MEF2C ubiquitination and degradation (Fig. 3, *A–F*, and S3, *A–D*), MEF2C phosphorylation (Fig. 4, *A–G*, and S4, *B* and *C*), MEF2C–DNA binding by PLA (Fig. 5, *A* and *B*), and RNA Poly II occupancy at the *CTNNA3* locus (Fig. 5*G*). Notably, we extensively used THP1^KO-fortilin^ cells for fortilin mutant analyses, which enabled evaluation of fortilin point mutants in the absence of confounding endogenous fortilin^WT^ (Figs. 2*H*, 3, *G* and *H*, 4, *H–J*, and 5, *G–I*).

The biological significance of the fortilin–MEF2C interaction in a whole-animal context remains unclear. MEF2C is essential for embryonic survival, as its constitutive deficiency results in early embryonic lethality because of its critical roles in a wide range of developmental programs in neuronal, skeletal, and cardiovascular tissues (58). Subsequent conditional KO studies have revealed additional *in vivo* functions of MEF2C, including but not limited to its involvement in endochondral bone ossification (70), hippocampal-dependent learning (71), hematopoietic differentiation (72, 73), and cardiovascular development (74). In adult mice, microglia-specific–inducible *Mef2c* deletion leads to an exaggerated proinflammatory response to tumor necrosis factor alpha challenge and a reduced preference for social interaction, which highlights the importance of the neuronal MEF2C pathway in immunoregulation and social behavior (75). Based on our finding that fortilin deficiency drastically reduces MEF2C expression in cells (Figs. 3, *A–D* and S3, *A–D*), we speculate that fortilin deficiency may initiate or exacerbate these MEF2C-dependent adverse phenotypes across various tissues in both developing and mature whole organisms, some of which may be mitigated by restoring fortilin expression.

## Experimental procedures

### Reagents and materials

#### Chemicals

We purchased CHX (catalog no.: C7698) and MG132 (catalog no.: M8699) from MilliporeSigma.

#### Recombinant proteins

We obtained the recombinant human proteins, MEF2A (catalog no.: TP312830; cMYC-FLAG-tagged), MEF2B (catalog no.: TP327214; cMYC-FLAG-tagged), MEF2C (catalog no.: TP320584; cMYC-FLAG-tagged), MEF2D (catalog no.: TP308748; cMYC-FLAG-tagged), and fortilin (catalog no.: TP301664; cMYC-FLAG-tagged) from OriGene and His_6_ - NQO2 (catalog no.: ab93933) from Abcam.

#### Plasmid vectors

We obtained the following mammalian expression vectors from GeneCopoeia.

1. pEZ-MEF2C^WT^-FLAG: pEZ-M39 containing the human *MEF2C* cDNA construct (GenBank accession ID: NM_00001131005.2) fused at the 3ʹ terminus to the FLAG-tag construct under the control of the cytomegalovirus promoter;
2. pEZ-MEF2C^M1^-FLAG: MEF2C deletion mutant of the pEZ-M39 vector containing amino acids 86 to 463 of MEF2C fused to a FLAG tag at the 3ʹ terminus; the M1 deletion removed the MADS-box and MEF2 domains from MEF2C;
3. pEZ-MEF2C^M2^-FLAG: MEF2C deletion mutant of the pEZ-M39 vector containing amino acids 271 to 463 of MEF2C fused to a FLAG tag at the 3ʹ terminus; this excluded the N-terminal and central domains and isolated the ERK5-binding domain;
4. pCS-fortilin^WT^-3×HA: The pCS-G0189-M91 containing the human *fortilin* (*TPT1*) cDNA construct (GenBank accession ID: NM_003295.4) fused at the 3ʹ terminus to the 3×HA-tag construct under the control of the cytomegalovirus promoter.
5. pCS-fortilin^D25A^-3×HA: The pCS-fortilin^WT^-3×HA vector in which Asp^25^ is replaced with Ala (D25A).

#### siRNA for gene silencing assays

We obtained the predesigned Accell SMART siRNA pools against human fortilin (siRNA^fortilin^) and MEF2C (siRNA^MEF^^2^^C^) from Dharmacon Horizon Discovery. siRNA^fortilin^ targeted the following four RNA sequences: GUGGCAAUUAUUUUGGAUC; GCAUGGUUGCUCUAUUGGA; UGACUGUGAUUUAUUUGGA; and CUUUAUUGGUGAAAACAUG. siRNA^MEF2C^ targeted the following four RNA sequences: GGAUUAUGGAUGAACGUAA; CUCUUGUCUAAUAUUCGUC; GCACUAGCACUCAUUUAUC; and CUGCCUUGUACUAAUGUUU. We used nontargeting pool siRNAs as the control (siRNA^control^).

#### Cell culture and cell lines

We obtained HEK293T and THP1 cell lines from the American Type Culture Collection. HEK293T cells were maintained in high-glucose Dulbecco's modified Eagle's medium (Corning, catalog no.: 10-013-CV) supplemented with 10% fetal bovine serum (Sigma, catalog no.: 21K475-A) and 1% antibiotic–antimycotic solution (GIBCO, catalog no.: 15240062) at 37°C in an atmosphere containing 5% CO_2_. THP1 cells were maintained in Roswell Park Memorial Institute 1640 medium (GIBCO, catalog no.: 61870127) supplemented with 10% fetal bovine serum and 1% antibiotic–antimycotic solution at 37°C in an atmosphere containing 5% CO_2_.

To obtain fortilin-deficient THP1 cells (THP1^KO-fortilin^), fortilin was knocked out by targeting exon 3 of the fortilin gene using the standard CRISPR–Cas9 system and the exon-targeting single-guide RNA (TTTCGGTACCTTCGCCCTCG). This procedure was performed by Synthego, and we characterized the cells as reported previously (9). THP1^WT-fortilin^ cells were used as the control.

### Methods

#### MST analysis

We performed MST using the NanoTemper Monolith NT.115 Pico system (NanoTemper Technologies GmbH) as described previously (9). Briefly, we first labeled rh-fortilin with the Monolith red–maleimide second-generation protein labeling kit (NanoTemper Technologies, catalog no.: MO-L014) according to the manufacturer’s protocol. We mixed 5 nM red–maleimide-labeled rh-fortilin with varying concentrations of MEF2C (range, 30 pM–250 nM; OriGene, catalog no.: TP320584) in PBS, loaded the mixture into Monolith NT.115 series premium glass capillaries (catalog no.: MO-K025), placed the capillaries in the MST system, and measured the pattern of thermophoresis of fortilin at 25°C using medium MST power and 1% LED excitation power. We first determined the normalized fluorescence value (F_norm_) by dividing the post-thermal signal (F_hot_) by the prethermal signal (F_initial_), where F_initial_ represents the fluorescence measured before the temperature gradient was applied and F_hot_ represents the fluorescence measured after heating-induced thermophoresis. We then calculated the dissociation constant (*K*_*d*_) using the NanoTemper analysis software after conducting three independent experiments. We repeated the same experiments for MEF2A (OriGene, catalog no.: TP312830), MEF2B (OriGene, catalog no.: TP327214), and MEF2D (OriGene, catalog no.: TP308748).

#### Real-time reverse transcription quantitative PCR

We performed real-time RT–qPCR assays as previously described (7, 9, 12, 13). Briefly, we harvested THP1^KO-fortilin^ and THP1^WT-fortilin^ cells, isolated total RNA using the GeneJET RNA Purification Kit (Thermo Fisher Scientific, catalog no.: K0731), and subjected 50 ng of total RNA to real-time RT–qPCR using the QuantiNova Probe RT–PCR kit (QIAGEN, catalog no.: 208354) with the following primer and probe sets (Integrated DNA Technologies).

1. Human *CTNNA3*—Forward: 5′-GAGGAGGAAATACGACCATCAC-3′, Reverse: 5′-GATAATCCGCTCTCGGTGTAAG-3′, Probe 5′-FAM-TGCTGGCGG/ZEN/ATTCTTTCAT-IBFQ-3′;
2. Human *fortilin* *(TPT1)—*Forward primer: 5ʹ-ATGACTCGCTCATTGGTGGAA-3ʹ, Reverse primer: 5ʹ-TGCTTTCGGTACCTTCGCCC-3ʹ, Probe: 5ʹ-FAM-TGCCTCCGC/ZEN/TGAAGGCCC-IBFQ-3ʹ;
3. Human *CD68*—Forward: 5ʹ -CCTCGCATGCTGATAACAATTC-3ʹ, Reverse: 5ʹ-CTCATTGAAGCGTGGGTTAAAG-3ʹ, Probe 5ʹ-FAM-CGGTGAAGC/ZEN/CCAATGCAAACAGAA-IBFQ-3ʹ;
4. Human *MAC2* *(LGALS3)*—Forward: 5ʹ-ACGCAACTGGCTCAAAGA-3′, Reverse: 5′-TCCCAAAGTGCTGGGATTAC-3′, Probe 5′-FAM-AAAGAAAGC/ZEN/CGGGCATGACGG-IBFQ-3′;
5. Human *VWF*—Forward primer: 5ʹ-GTACAGCTTTGCGGGATACT-3ʹ, Reverse primer: 5′-GCTCACTCTCTTGCCATTCT-3ʹ, Probe: 5ʹ-FAM-TGCCAGAAA/ZEN/CGCTCCTTCTCGATT-IBFQ-3ʹ;
6. Human *tissue factor (TF)*—Forward primer: 5ʹ-CCGAA AGTTAACCGGAAGAG-3ʹ, Reverse primer: 5ʹ-CCACAGCTCCAATGATGTAGAA-3ʹ, Probe: 5ʹ-FAM-CGGTAGAGT/ZEN/GTATGGGCCAGGAGA-IBFQ-3ʹ;
7. Human *NOS3*—Forward primer: 5ʹ-CCGGAACAGCACAAGAGTTA-3ʹ, Reverse primer: 5ʹ-GTCTGTGTTACTGGACTCCTTC-3ʹ, Probe: 5ʹ-FAM-TCTGAGCAG/ZEN/GAGATGCTGTTGAAGC-IBFQ-3ʹ; and
8. Human 18S rRNA—Forward: 5′-CTGAGAAACGGCTACCACATC-3′, Reverse: 5′-GCCTCGAAAGAGTCCTGTATTG-3′, Probe 5′-JOEN-AAATTACCC/ZEN/ACTCCCGAC-IBFQ-3′;

where FAM = carboxyfluorescein, ZEN = an internal quencher to enhance the quenching activity of the 3ʹ quencher IBFQ (IDT), and JOEN = 6-carboxy-4′,5′-dichloro-2′,7′-dimethoxyfluorescein. We used the 2^–ΔΔCT^ method to calculate the expression levels (AU) of a gene in question relative to the 18S rRNA levels in the sample.

#### Western blot analyses

SDS-PAGE and Western blot analyses were performed as described previously (7, 9, 13). Briefly, protein concentrations were measured using a Protein Assay Dye Reagent Concentrate (Bio-Rad, catalog no.: 5000006), and an appropriate amount of proteins was loaded into each lane of the 10% polyacrylamide gel for electrophoresis. When appropriate, 0.5% (v/v) 2,2,2-trichloroethanol was added to a polyacrylamide gel before polymerization to quantify total proteins loaded on each lane of the gel. After standard SDS-PAGE, the 2,2,2-trichloroethanol-containing gel was ultraviolet-irradiated on the Bio-Rad ChemiDoc MP Imaging System for 2 min, its image was electronically captured, and the cumulative band densities were calculated to assess loading conditions as described previously (7). The proteins resolved on the gel were then blotted onto a nitrocellulose membrane (pore size = 0.45 μm; Bio-Rad, catalog no.: 1620115) using the Trans-Blot Turbo semidry transfer device (Bio-Rad). The membrane was then blocked with 3% skim milk (Bio-Rad, catalog no.: 1704270) in Tris-buffered saline with Tween overnight, incubated with a primary Ab, and then incubated with an appropriate horseradish peroxidase–labeled secondary Ab as described below. Developed images were electronically captured using the ChemiDoc MP Imaging System, and the signal intensities of protein bands were quantified using ImageJ software (National Institutes of Health).

The following primary antibodies were used.

1. Mouse anti-fortilin mAb (1:2500 dilution; Abnova Corporation, catalog no.: H00007178-M03);
2. Rabbit anti-MEF2C mAb (1:1000 dilution; Abcam, catalog no.: ab211493; clone no.: EPR19089-202);
3. Mouse anti-MEF2C mAb (1:500 dilution; Abcam, catalog no.: ab118406; clone no.: OTI4B10);
4. Rabbit anti-MEF2A mAb (1:1000 dilution; Abcam, catalog no.: ab76063; clone no.: EP1706Y);
5. Rabbit anti-MEF2B mAb (1:1000 dilution; Abcam, catalog no.: ab242058; clone no.: EPR22193-26);
6. Rabbit anti-MEF2D mAb (1:1000 dilution; Abcam, catalog no.: ab282731; clone no.: EPR24993-10);
7. Rabbit anti-fortilin mAb (recombinant; 1:3000 dilution; Abcam, catalog no.: ab133568);
8. Mouse anti-FLAG mAb (1:1000 dilution; Sigma, catalog no.: F1804); and
9. Mouse α-His6 mAb (1:1000 dilution; Abcam, catalog no.: ab18184).

The following secondary antibodies were used.

1. IRDye 800CW goat anti-rabbit pAb (1:5000 dilution; LI-COR, catalog no.: D101324-25);
2. IRDye 680CW goat anti-mouse pAb (1:5000 dilution; LI-COR, catalog no.: D20920-15).

#### JESS protein analyses

We used the JESS Simple Western System (ProteinSimple) to assess the expression levels of FLAG-tagged MEF2C and phospho-MEF2C (Ser^59^) in a highly quantitative fashion as previously described (9). Briefly, we first generated TCLs in lysis buffer (0.1% NP-40, 100 mM Tris–HCl [pH 6.8], 150 mM NaCl, 1 mM EDTA), protease inhibitor cocktail (Thermo Fisher Scientific, catalog no.: 87786), and PhosSTOP phosphatase inhibitor (Roche, catalog no.: 0490684500) at the concentration of 1 μg/μl. We then mixed the Fluorescent 5x Master Mix (ProteinSimple, catalog no.: PS-FL01-8) and the sample at a ratio of 1:4, resulting in the final sample concentration of 0.8 μg/μl. After boiling the mixture at 95 °C for 5 min, we centrifuged it at 500*g* for 5 min and loaded it into an appropriate well within the prefilled plate for the 12 to 230 kDa capillary (ProteinSimple, catalog no.: SM-W004-1).

We used the following primary antibodies.

1. Rabbit anti-FLAG pAb (1:50 dilution; Sigma, catalog no.: F7425);
2. Rabbit anti-MEF2C pAb (1:100 dilution; Abcam, catalog no.: ab227085);
3. Rabbit anti-phospho-MEF2C (S59) pAb (1:50 dilution; Abcepta, catalog no.: AP3349a);
4. Rabbit anti-fortilin mAb (recombinant; 1:100 dilution; Abcam, catalog no.: ab133568, clone no.: EPR5540); and
5. Rabbit anti-CTNNA3 mAb (recombinant; 1:200 dilution; Abcam, catalog no.: ab184916, clone no.: EPR18307).

We used the anti-Rabbit secondary horseradish peroxidase Ab (ProteinSimple, catalog no.: 042-206) as the secondary Ab and Luminol–Peroxide Mix (Luminol-S, ProteinSimple, catalog no.: 043-311; Peroxide, ProteinSimple, catalog no.: 043-379) to visualize the chemiluminescent signals from bound antibodies. We then quantified total proteins loaded in each capillary using the total protein detection module (ProteinSimple, catalog no.: DM-TP01). We quantified the digitized chemiluminescence signals for both specific and total proteins and quantified them using Compass Simple Western software (version 6.3.0, ProteinSimple). Specifically, we calculated the protein expression index (AU) by dividing the area under the curve of a protein of interest by the total proteins loaded in the same capillary (“in-capillary normalization”).

#### Analysis of CTNNA3 in THP1 cells overexpressing MEF2C-binding–deficient fortilin (fortilin^D25A^)

We plated 2 × 10^4^THP1^KO-fortilin^ cells per well in 24-well plates, incubated the cells overnight, and transiently transfected them using Lipofectamine 3000 (Invitrogen; catalog no.: 3000-075) with pCS-fortilin^WT^-3×HA or pCS-fortilin^D25A^-3×HA. After 48 h of incubation, we washed the cells with PBS and harvested them in lysis buffer (0.1% NP-40, 100 mM Tris–HCl [pH 6.8], 150 mM NaCl, and 1 mM EDTA) supplemented with the protease inhibitor cocktail and PhosSTOP phosphatase inhibitor.

We subjected 4 μg of TCLs to analysis using the JESS Western blot system and detected plasmid-derived fortilin-HA using a rabbit anti-HA mAb (1:100 dilution; Cell Signaling Technology; catalog no.: 3724S) and CTNNA3 using a rabbit anti-CTNNA3 mAb (1:200 dilution; Abcam; catalog no.: ab184916). We visualized total protein as described above and used it as a loading control. We quantified CTNNA3 expression using Compass software (version 6.3.0) by calculating the area under the curve of the CTNNA3 peak normalized to total protein signal. We expressed results in AU.

#### *In vitro* and *in vivo* co-IP–Western blot analyses

For *in vitro* co-IP Western blot analysis to evaluate the direct interaction between fortilin and MEF2C, we added human recombinant fortilin-FLAG (OriGene; TP301664), MEF2C-FLAG (OriGene; TP320584), His_6_ - NQO2 (Abcam; ab93933) in lysis buffer (10 mM Tris, pH 7.4, 100 mM NaCl, 0.01% NP-40, 2 mM PMSF [Thermo Fisher Scientific; catalog no.: 36978]), and the protease inhibitor cocktail and incubated the mixture at room temperature for 2 h with rotation in the presence of rabbit anti-fortilin mAb (Abcam; ab133568). Next, we added 50 μl of Dynabeads M-280 Sheep Anti-Rabbit IgG (Invitrogen, catalog no.: 01266125) and further incubated the mixture for 1 h at room temperature with gentle rotation. After four washes with wash buffer (10 mM Tris, pH 7.4, 100 mM NaCl, 0.3% NP-40, 2 mM PMSF, and protease inhibitor cocktail), we eluted the immunoprecipitated proteins from the beads in 2x SDS gel loading buffer (50 mM Tris–HCl, pH 6.8, 1% 2-mercaptoethanol, 1% SDS, 25 mM EDTA, 0.01% bromophenol blue, and 10% glycerol) and subjected the eluents to SDS-PAGE and Western blotting (Fig. S1*G*).

For *in vivo* co-IP Western blot analysis to evaluate the interaction between fortilin and MEF2C and its isoforms in the cell, we first lysed 293T cells in 500 μl of lysis buffer (PBS, 0.001% NP-40, and protease inhibitor cocktail) on ice, followed by brief sonication (Bioruptor, Diagenode). We centrifuged the lysates at 16,000*g* at 4 °C for 10 min and generated the TCL. We used 10% of the TCL (50 μl) for input analyses.

For forward *in vivo* co-IP, we incubated 200 μl of TCLs with a rabbit anti-fortilin mAb (Abcam, catalog no.: ab133568) overnight at 4 °C with rotation. On the following day, we added 50 μl of Dynabeads M-280 sheep anti-rabbit IgG suspension (Invitrogen, catalog no.: 01266125), which we had preblocked with 3% bovine serum albumin in wash buffer (PBS containing 0.0001% NP-40 and the protease inhibitor cocktail). We incubated the mixture for 1 h at 4 °C, washed the beads three times with wash buffer, eluted bound proteins in 2x SDS gel loading buffer, and subjected the eluates to SDS-PAGE and Western blot analyses using (a) anti-fortilin mAb to verify the successful fortilin IP and (b) mAbs against MEF2A (Abcam, catalog no.: ab76063), MEF2B (Abcam, catalog no.: ab242058), MEF2C (Abcam, catalog no.: ab118406), and MEF2D (Abcam, catalog no.: ab282731) to assess the presence of MEF2C and other MEF2 isoforms coimmunoprecipitated with fortilin (Fig. 1, *F–I*).

For reverse *in vivo* co-IP, we followed the same procedure as for forward IP, except for (a) incubating the lysate with a rabbit anti-MEF2C mAb (Abcam, catalog no.: ab211493) and (b) abelled the eluates by Western blot analyses using (a) anti-MEF2C mAb (Abcam, catalog no.: ab211493) to verify the successful IP of MEF2C and (b) anti-fortilin mAb (Abnova, catalog no.: ABIN521072) to evaluate the presence of fortilin coimmunoprecipitated by MEF2C (Fig. 1*J*).

To evaluate the interaction of fortilin with MEF2C^WT^ and its deletion mutants—MEF2C^M1^ (MEF2C mutant consisting of amino acids 86–463) and MEF2C^M2^ (MEF2C mutant consisting of amino acids 271–463)—we first transiently transfected 293T cells with either the pEZ-M39-MEF2C^WT^-FLAG, pEZ-MEF2C^M1^-FLAG, or pEZ-MEF2C^M2^-FLAG mammalian expression plasmid using Lipofectamine 3000 (Invitrogen, catalog no.: 3000-075). We then lysed the cells in lysis buffer (PBS, 0.001% NP-40, and protease inhibitor cocktail) and PhosSTOP phosphatase inhibitor, incubated the TCL with mouse anti-FLAG M2 magnetic beads (Sigma–Aldrich, catalog no.: SIAL-M8823) for 3 h at 4 °C, washed the beads with wash buffer (PBS, 0.0001% NP-40, protease inhibitor cocktail, and PhosSTOP phosphatase inhibitor), eluted the bound proteins in 2x SDS gel loading buffer, and subjected them to SDS-PAGE and Western blot analyses (Fig. 2*B*).

To evaluate the interaction between MEF2C and fortilin^D25A^—a point mutant of fortilin in which Asp^25^ has been mutated to Ala—we transiently transfected THP1^KO-fortilin^ cells with pCS-fortilin^WT^-3×HA or pCS-fortilin^D25A^-3×HA, or control plasmid using Lipofectamine 3000 (Invitrogen, catalog no.: 3000-075). We lysed the cells in lysis buffer (PBS, 0.001% NP-40, protease inhibitor cocktail, and PhosSTOP phosphatase inhibitor). We incubated the lysates with anti-HA magnetic beads (Pierce–Thermo Fisher Scientific, catalog no.: 88836) for 3 h at 4 °C, washed the beads with wash buffer (PBS, 0.0001% NP-40, protease inhibitor cocktail, and PhosSTOP phosphatase inhibitor), eluted bound proteins in 0.1 × sample buffer (ProteinSimple, catalog no.: 042-195), subjected them to the JESS Western blot system using rabbit anti-MEF2C pAb (1:100 dilution; Abcam, catalog no.: ab227085) and rabbit anti-HA mAb (1:100 dilution; Cell Signaling Technology; catalog no.: 3724S), and analyzed the data as described in the JESS protein analyses section above.

#### Proximity ligation assay

We performed PLA using a commercially available kit (Sigma, Duolink series) as previously described (9, 12, 13) to verify the presence of an interaction between fortilin and MEF2C (Fig. 1, *K* and *L*) or dsDNA (Fig. 5, *A* and *B*). Briefly, we seeded THP1 cells on a 4-chamber slide (Thermo Fisher Scientific, catalog no.: 154526), treated them with 100 ng/ml of phorbol 12-myristate 13-acetate (Sigma, catalog no.: P8139) for 96 h, fixed them with 4% paraformaldehyde for 20 min, permeabilized them with 0.1% Triton X-100 in PBS for 15 min, and blocked them with Duolink Blocking solution (Sigma) at 37 °C for 1 h. To visualize the fortilin–MEF2C interaction, we incubated the cells with rabbit anti-fortilin mAb (1:200 dilution; Abcam, catalog no.: ab133568) and mouse anti-MEF2C mAb (1:200 dilution; Abcam, catalog no.: ab118406). To visualize the MEF2C–dsDNA interaction, we incubated the cells with rabbit anti-MEF2C pAb (1:200 dilution; Abcam, catalog no.: ab227085) and mouse anti-dsDNA (HYB331-01) mAb (1:100 dilution; Santa Cruz Biotechnology, catalog no.: sc-58749) overnight at 4 °C. The next morning, after washing with wash buffer (Sigma, Duolink, catalog no.: DUO82049), we incubated the cells with the Duolink PLA probes, which were anti-mouse and anti-rabbit Abs conjugated to oligonucleotides (Sigma, Duolink series; PLA probe anti-mouse MINUS, catalog no.: DUO92004 and PLA probe anti-Rabbit PLUS, catalog no.: DUO92002) for 1 h at 37 °C. We then added ligase and two connector oligonucleotides to the solution so that the connector oligonucleotides would hybridize to the two PLA probes and join them into a closed circle if they were in close proximity (30 nm) to each other. Finally, we added Texas Red–abelled oligonucleotides that would hybridize to the rolling circle amplification products in the solution (Sigma, Duolink series; PLA Detection Kit Red, catalog no.: DUO92008) and applied a coverslip using mounting media containing 4′,6-diamidino-2-phenylindole (Sigma, Duolink series, catalog no.: DUO82040). We visualized the signals using a Nikon A1R confocal microscope and captured images using NIS-Elements AR image acquisition software (version 5.11.01; Nikon). We calculated the PLA interaction index by dividing the number of PLA puncta by the number of nuclei within a field of view and expressed the value in AU. We analyzed three fields of view per experiment to derive a PLA interaction index for each experiment. We performed three independent experiments to compare the two groups.

### *In vivo* MEF2C ubiquitin–proteasome degradation assays

#### MEF2C degradation assays

To evaluate the degradation of plasmid-derived, FLAG-tagged MEF2C in the presence and absence of fortilin, we plated 2 × 10^4^ THP1^WT-fortilin^ and THP1^KO-fortilin^ cells (9) in each well of 24-well plates, incubated them overnight, transiently transfected the cells using Lipofectamine 3000 (Invitrogen, catalog no.: 3000-075) with pEZ-MEF2C^WT^-FLAG, and incubated them for 48 h. Subsequently, we exchanged the culture media for new media containing 100 μg/ml CHX with or without a proteasome inhibitor (20 μM MG132 for Fig. S3, *A–D*; 10 nM carfilzomib [Sigma; catalog no.: F4770] for Fig. 3, *A–D*) and incubated the cells at 37°C in an atmosphere containing 5% CO_2_. We harvested the cells at 0, 4, 8, 12, and 24 h after the media exchange into lysis buffer (0.1% NP-40, 100 mM Tris–HCl [pH 6.8], 150 mM NaCl, 1 mM EDTA, protease inhibitor cocktail, and PhosSTOP phosphatase inhibitor) and subjected 4 μg of lysates to JESS as previously described (9). We detected plasmid-derived MEF2C-FLAG in each capillary using anti-FLAG pAb (1:50 dilution; Sigma, catalog no.: F7425). We also visualized total proteins and used them as a loading control. We calculated the MEF2C expression index as the area under the curve of the MEF2C-FLAG peak divided by that of total proteins and expressed it in AU, using Compass software (version 6.3.0) as described above.

#### MEF2C ubiquitination assays

To evaluate the impact of fortilin deficiency on MEF2C ubiquitination, we performed a ubiquitination assay as previously described (9), with modifications. We first seeded THP1^WT-fortilin^ (WT) and THP1^KO-fortilin^ (KO) cells (9) and treated them with 10 nM carfilzomib (Sigma), a proteasome inhibitor, and 10 μM ML364 (Sigma; catalog no.: SML1920), a deubiquitinase inhibitor, for 12 h. We then lysed the cells in buffer containing 1% SDS, 50 mM Tris–HCl (pH 7.5), 150 mM NaCl, protease inhibitor cocktail, and PhosSTOP phosphatase inhibitor. We briefly sonicated the lysates, cleared them by centrifugation, and diluted them 10-fold with IP buffer (PBS, 0.001% NP-40, and protease inhibitor cocktail). We incubated the diluted lysates overnight at 4 °C with rabbit anti-MEF2C mAb (Abcam; catalog no.: ab211493; clone no.: EPR19089-202), followed by incubation with Dynabeads M-280 sheep anti-rabbit IgG (Invitrogen; catalog no.: 01266125) for 1 h at 4 °C. We then washed the beads three times with wash buffer (PBS, 0.0001% NP-40, and protease inhibitor cocktail) and eluted bound proteins in 0.1 × sample buffer (ProteinSimple; catalog no.: 042-195) for JESS analysis. We analyzed the eluates using the JESS system with rabbit anti-MEF2C polyclonal Ab (1:50 dilution; Abcam; catalog no.: ab227085) and mouse anti-ubiquitin mAb (1:50 dilution; Cell Signaling Technology; catalog no.: 3936S; clone no.: P4D1). Using Compass software, we calculated the MEF2C ubiquitination index by dividing the total signal intensity of ubiquitinated MEF2C immunoprecipitated by anti-MEF2C Ab by the signal intensity of unmodified MEF2C present in TCLs prior to IP. We expressed results in A.U.

#### MEF2C degradation assay using the MEF2C-binding–deficient mutant of fortilin (fortilin^D25A^)

To evaluate whether MEF2C-binding–deficient fortilin mutants affect MEF2C degradation, we plated 2 × 10^4^ THP1^KO-fortilin^ cells per well in 24-well plates and incubated them overnight. We then transiently transfected the cells with pCS-fortilin^WT^-3xHA or pCS-fortilin^D25A^-3×HA using Lipofectamine 3000 (Invitrogen; catalog no.: 3000-075). After 48 h, we washed the cells with PBS, replaced the culture medium with fresh medium containing 100 μg/ml CHX in the presence or the absence of 10 nM carfilzomib, and incubated the cells at 37°C in a humidified atmosphere containing 5% CO_2_. We harvested the cells 12 h after medium exchange and lysed them in lysis buffer containing 0.1% NP-40, 100 mM Tris–HCl (pH 6.8), 150 mM NaCl, 1 mM EDTA, protease inhibitor cocktail, and PhosSTOP phosphatase inhibitor. We then subjected 4 μg of each lysate to analysis using the JESS system, as previously described (9). We detected plasmid-derived fortilin-HA using rabbit anti-HA mAb (1:100 dilution; Cell Signaling Technology; catalog no.: 3724S) and detected MEF2C using rabbit anti-MEF2C polyclonal Ab (1:50 dilution; Abcam; catalog no.: ab227085). We visualized total protein in each capillary and used it as a loading control. Using Compass software (version 6.3.0), we calculated the MEF2C expression index as the area under the curve of the MEF2C peak normalized to total protein signal. We expressed results in AU.

### MEF2C phosphorylation assay

#### Evaluation of MEF2C phosphorylation status using Pro-Q Diamond

To evaluate the impact of fortilin on the phosphorylation of MEF2C, we transiently transfected THP1^WT-fortilin^ and THP1^KO-fortilin^ cells with pEZ-MEF2C^WT^-FLAG using Lipofectamine 3000, incubated the cells for 48 h, and lysed them in lysis buffer (PBS, 0.001% NP-40, protease inhibitor cocktail, and PhosSTOP phosphatase inhibitor). We incubated the TCL with anti-FLAG M2 magnetic beads (Sigma, catalog no.: SIAL-M8823-1 ML) for 3 h at 4 °C, washed it with wash buffer (PBS, 0.0001% NP-40, and protease and phosphatase inhibitors), eluted the proteins in elution buffer (0.1 M glycine–HCl, pH 2.5, protease inhibitor cocktail, and PhosSTOP phosphatase inhibitor), added the SDS loading buffer, and heated the eluates at 95 °C for 5 min. We performed SDS-PAGE on these samples along with PeppermintStick Phosphoprotein Molecular Weight Standards (ThermoFisher, catalog no.: P27167), fixed the gel in fix solution (50% methanol, 10% acetic acid), stained it with Pro-Q Diamond (Thermo Fisher Scientific, catalog no.: P33300) for 70 min to visualize phosphorylated MEF2C, and captured the image of the stained gel using the ChemiDoc MP Imaging System (illumination source: Epi-green, 520–545 nm excitation; filter: 590/110 nm standard filter). We subsequently stained the gel overnight with SYPRO Ruby (Thermo Fisher Scientific, catalog no.: S12000) to visualize total MEF2C and captured the image of the stained gel using the ChemiDoc MP Imaging System (illumination source: Trans-UV, 302 nm excitation; filter: 590/110 nm standard filter).

#### Evaluation of MEF2C-Ser^59^ phosphorylation by JESS

We evaluated Ser^59^ phosphorylation of endogenous MEF2C in the presence and absence of fortilin using the JESS system (ProteinSimple) as described above under JESS protein analyses. To assess Ser^59^ phosphorylation of immunoprecipitated MEF2C in the presence and absence of fortilin, we seeded THP1^WT-fortilin^ and THP1^KO-fortilin^ cells, treated the cells with carfilzomib (10 nM; Sigma, catalog no.: F4770), a proteasome inhibitor, for 12 h, and prepared TCLs using a lysis buffer consisting of PBS, 0.001% NP-40, protease inhibitor cocktail, and PhosSTOP phosphatase inhibitor. We incubated the TCLs overnight at 4 °C with a rabbit anti-MEF2C mAb (Abcam, catalog no.: ab211493). The following day, we washed the immunocomplexes with wash buffer (PBS, 0.0001% NP-40, protease inhibitor cocktail, and PhosSTOP phosphatase inhibitor) and eluted bound proteins in 0.1 x sample buffer (ProteinSimple, catalog no.: 042-195) for JESS analysis. We subjected the eluents to JESS using the following antibodies: rabbit anti-MEF2C pAb (1:100 dilution; Abcam, catalog no.: ab227085) to detect total MEF2C; rabbit anti-phospho-MEF2C (Ser^59^) pAb (1:50 dilution; Abcepta, catalog no.: AP3349a) to detect Ser^59^ phosphorylation; and rabbit anti-fortilin mAb (1:100 dilution; Abcam, catalog no.: ab133568). We also visualized total protein and used it as a loading control. We used Compass software (version 6.3.0) to calculate the MEF2C phosphorylation index, defined as the ratio of the area under the curve of the phospho-MEF2C signal to that of the total MEF2C signal. We expressed results in AU.

To evaluate the role of fortilin–MEF2C binding in MEF2C phosphorylation, we plated 2 × 10^4^ THP1^KO-fortilin^ cells per well in 24-well plates, incubated them overnight, and transiently transfected the cells with pCS-fortilin^WT^-3×HA and pCS-fortilin^D25A^-3×HA, using Lipofectamine 3000 (Invitrogen, catalog no.: 3000-075). Fortilin^D25A^ does not bind MEF2C (Fig. 2*H*). We incubated the cells for 48 h in the presence or the absence of carfilzomib (10 nM; Sigma), a proteasome inhibitor, washed them with PBS, and prepared TCLs in lysis buffer containing 0.1% NP-40, 100 mM Tris–HCl (pH 6.8), 150 mM NaCl, 1 mM EDTA, protease inhibitor cocktail, and PhosSTOP phosphatase inhibitor. We subjected 4 μg of the TCL to JESS analysis using the following antibodies: rabbit anti-phospho-MEF2C (Ser^59^) pAb (1:50 dilution; Abcepta) to detect Ser^59^-phosphorylated-MEF2C; rabbit anti-MEF2C pAb (1:100 dilution; Abcam) to detect total MEF2C; and rabbit anti-HA mAb (1:100 dilution; Cell Signaling Technology; catalog no.: 3724S) to detect plasmid-derived HA-tagged fortilin. We also visualized total protein and used it as a loading control. We calculated the MEF2C phosphorylation index using Compass software (version 6.3.0), as described above.

#### Dual luciferase assay

We transiently transfected 293T cells with the CTNNA3 firefly luciferase vector (pGL-PCTNNA3-Luc) from Dr Geert Berx’s laboratory (Ghent University, Belgium) along with the Renilla luciferase control reporter vector (pGL4.74 [hRluc/TK], Promega, part no.: E692A), which allowed us to normalize firefly luciferase activities according to transfection efficiency. After 48 h, we subjected the cells to the Dual-Glo-Luciferase Assay System (Promega) according to the manufacturer’s instructions. We calculated relative luciferase activity (firefly luciferase unit/Renilla luciferase unit) by dividing firefly luciferase units by Renilla luciferase units and expressed the results in AU.

#### TBP and Pol II ChIP

We performed TBP and Pol II ChIP assays to assess the recruitment of TBP and Pol II, respectively, to the TSS of the *CTNNA3* gene (NM_001127384.3). We used the *CD68* gene as a positive control and the vWF gene as a negative control, based on our previous work showing that fortilin increases *CD68* mRNA levels without altering *VWF* mRNA levels (28). We designed primers for ChIP–qPCR by first identifying the TSS of each gene using RefSeq Curated/MANE Select annotations (76). We then retrieved DNA sequences spanning −100 to +100 base pairs (bp) relative to the annotated TSS from the UCSC Genome Browser (Human/GRCh38) (77) and designed primers using Primer3web (version 4.1.0; https://primer3.ut.ee/) (78). For *CTNNA3*, which is encoded on the negative strand, we identified the TSS at chr10:67,665,660 and defined the corresponding ChIP target region as chr10:67,665,560–67,665,760 (TSS ± 100 bp). Similarly, we defined the ChIP target regions for *CD68* and *VWF* as chr17: 7,579,538–7,579,738 and chr12: 5,948,777–5,948,977, respectively.

We used the following primer pairs for ChIP-qPCR.

• Human *CTNNA3* promoter (100 bp amplicon)—Forward: TCATGGCTCACAATTTGGCTTAT; Reverse: GCTTGGGCCACTGTGTCC
• Human *CD68* promoter (201 bp amplicon)—Forward: TGAGTCAGGCTGTGGGTGG; Reverse: CTTCCTCCTTACCTGCCAGT
• Human *VWF* promoter (141 bp amplicon)—Forward: CTTCCTTCCTCAGAACCGGT; Reverse: GTTCTGCTCTTGTGCCCTTC.

We performed ChIP in quadruplicates using the MAGnify ChIP System (Invitrogen; catalog no.: 492024) according to the manufacturer’s instructions. Briefly, we crosslinked THP1^WT-fortilin^ and THP1^KO-fortilin^ cells with 1% formaldehyde and quenched the reaction with 1.25 M glycine. We then generated TCLs using lysis buffer provided in the kit and sheared chromatin by sonication. After centrifugation, we collected the supernatant containing fragmented chromatin. Next, we immunoprecipitated TBP and Pol II in the supernatant using an anti-TBP polyclonal Ab (Abcam, catalog no.: ab63766) and anti-Poly II mAb (Active Motif, clone no.: 4H8; catalog no.: 39097) coupled to Dynabeads Protein A/G beads and incubated the reaction mixture at 4 °C for 2 h. We used mouse normal IgG coupled to Dynabeads Protein A/G beads as a control. We washed the beads with IP buffer 1 and IP buffer 2 according to the manufacturer’s instructions. We then eluted the immunoprecipitated chromatin, reversed crosslinks, treated samples with proteinase K, and purified DNA using the magnetic bead–based purification system provided in the kit. We analyzed purified DNA by qPCR, using the above primer sets and PowerUp SYBR Green Master Mix for qPCR (Thermo Fisher Scientific; catalog no.: A25742) on a QuantStudio 3 Real-Time PCR System (Thermo Fisher Scientific). We calculated the TBP occupancy index at the *CTNNA3* locus as the fold enrichment of the *CTNNA3* TSS fragment immunoprecipitated with the α-TBP Ab relative to that immunoprecipitated by normal IgG, using Ct values obtained for *CTNNA3* (Ct-CTNNA3) and control normal IgG control (Ct-IgG) according to the following equation:

TBP occupancy index for *CTNNA3* = fold enrichment^CTNNA3^ = 2^(Ct-IgG – Ct-CTNNA3)^.

We calculated the TBP occupancy index as fold enrichment for *CD68* and *VWF* in the same manner and expressed all indices in A.U. We calculated Pol II occupancy indices for *CTNNA3*, *CD68*, and *VWF* using the same approach.

#### Subcellular fractionation

To obtain nuclear and cytoplasmic protein fractions, we used the Nuclear Extract Kit (Active Motif, catalog no.: 40010) following the manufacturer’s instructions. Briefly, 293T cells grown to confluence in a 10 cm dish (∼8.8 × 10^6^ cells) were washed twice with ice-cold PBS containing phosphatase inhibitors, gently scraped, and collected by centrifugation at 16,000*g* for 5 min at 4 °C. The cell pellet was resuspended in 500 μl 1× hypotonic buffer, incubated on ice for 15 min, and lysed with 25 μl detergent by vortexing for 10 s. The lysate was centrifuged at 16,000*g* for 30 s at 4 °C to obtain the cytoplasmic fraction. The remaining pellet was resuspended in complete lysis buffer (44.5 μl lysis buffer AM1, 0.5 μl protease inhibitor cocktail, and 5 μl 10 mM dithiothreitol) with 2.5 μl detergent, incubated on ice for 30 min on a rocking platform, vortexed for 30 s, and centrifuged at 16,000*g* for 10 min at 4 °C. The nuclear fraction was directly used for SDS-PAGE and Western blot analyses. The following primary antibodies were used:

1. Mouse anti-MEF2C mAb (1:500 dilution; Abcam, catalog no.: ab118406);
2. Mouse anti-fortilin mAb (1:2500 dilution; Abnova, catalog no.: H00007178-M03).

### Sequence analyses of MEF2A, MEF2B, MEF2C, and MEF2D

We used the following human protein sequences from the database at the National Center for Biotechnology Information for the analyses.

1. MEF2A, NP_001124398.1;
2. MEF2B, NP_001139257.1;
3. MEF2C, NP_001124477.1; and
4. MEF2D, NP_005911.1.

As we previously described (9), we first determined percent (%) identify matrices using the Simple Phylogeny platform available on the European Molecular Biology Laboratory–European Bioinformatics Institute website (URL: https://www.ebi.ac.uk/jdispatcher/phylogeny/simple_phylogeny) and by entering the protein sequences of these four MEF2C family proteins. We then visualized them by generating a phylogenetic tree using the same platform and calculated the evolutionary distances among these four catenins. Finally, we used SnapGene (version 7.2; Dotmatics) to align the protein sequences of MEF2A, MEF2B, MEF2C, and MEF2D.

#### Identification of MEF2C binding sites in the CTNNA3 promoter region

We evaluated the potential MEF2C binding sites within the *CTNNA3* promoter region using the JASPAR 2024 database (55) (URL: https://jaspar.elixir.no/). We obtained the sequence for the *CTNNA3* promoter region from GenBank (accession no.: AF361938) and as previously described by Vanpoucke *et al.* (26). We scanned the FASTA-formatted promoter sequence (from −3000 to 0 relative to the TSS) for the MEF2C binding motif (matrix ID: MA0497.1 and MA0497.2) on the JASPAR platform by applying a score threshold of >90%. We identified just one predicted sequence: ATAAAAATACA (positions from −976 to −966, relative score = 91.6%).

#### Molecular docking simulation

To assess the physical interactions between fortilin and members of the MEF2 family, we performed molecular docking simulations using the HADDOCK 2.4 platform (https://rascar.science.uu.nl/haddock2.4/) (35, 79). We obtained protein structures from the PDB: fortilin (PDB ID: 5O9M), MEF2A (PDB ID: 1C7U), MEF2B (PDB ID: 1N6J), MEF2C (PDB ID: 5F28), and MEF2D (PDB ID: 8C84). We prepared them using PyMOL (Schrödinger, Inc; https://pymol.org). We then performed energy minimization using UCSF Chimera (80) and subsequently uploaded the structures to the HADDOCK platform.

We carried out the HADDOCK docking protocol in three stages: (i) randomization of orientations followed by rigid-body energy minimization (it0), (ii) semiflexible simulated annealing in torsion-angle space (it1), and (iii) a final short energy minimization step. We defined passive residues using the HADDOCK server as solvent-accessible amino acids neighboring active residues within 6.5 Å. We set the initial cutoff for the restraint-solvating method at 5.0 Å, such that all water molecules located farther from a highly occurring water-solvated residue were removed during the generation of the initial solvation shell. We subsequently clustered the generated structures based on the fraction of common contacts using a minimum cluster size of four and an RMSD cutoff of 7.5 Å. We ranked representative clusters according to the average HADDOCK score of the four lowest-energy structures using default weighting parameters (https://rascar.science.uu.nl/haddock2.4/settings). We assessed model quality using CAPRI criteria (81), calculating ligand RMSD after fitting the smaller protein onto the interface of the larger protein. We analyzed intermolecular contacts using a distance threshold of 4.5 Å. We visualized and analyzed representative structures from the top-ranked cluster using PyMOL and custom PyMOL scripts for interface characterization. For molecular dynamics simulations, we utilized the WebGRO server (https://webgro.uams.edu), which runs on the GROMACS software package (82). We performed molecular dynamics simulations using the CHARMM27 force field, following procedures described previously (83). Resulting complexes were then visualized using both PyMOL and Maestro (Schrödinger, Inc), and molecular surfaces were rendered to examine docking interfaces.

#### Statistics and reproducibility

The degree of the spread of data was expressed by the standard deviation. A two-tailed unpaired *t* test was used to compare the means of two groups. To compare the means of three or more groups, we used two-way ANOVA with Tukey–Kramer’s pairwise comparison. *p* < 0.05 was considered to be statistically significant. Prism software 10.1.2 (GraphPad Software) was used for visualization and statistical analyses. The numbers of biological replicates used in each experiment and the statistical test used to analyze the particular experiment are provided in the article. Normality of the data was assessed using the Shapiro–Wilk test (α = 0.05), and unless otherwise stated, the data were found to be normally distributed.

## Data availability

The authors declare that the data supporting the findings of this study are available within the article and its supporting information files. All relevant data are available from the authors upon request.

## Supporting information

This article contains supporting information.

## Conflict of interest

K. F. is an officer and equity holder of Fortiscience, Inc, a university spin-off focused on the development of fortilin-targeted therapeutics. All other authors declare no competing financial or nonfinancial interests. This study was conducted as fundamental research, and the findings were reached independently of any commercial interests. The funders had no role in study design, data collection and analysis, decision to publish, or preparation of the article.
