# Supplementary material for: Fortilin binds and stabilizes MEF2C, activates it through phosphorylation, and drives transcription of the cell structural and survival protein CTNNA3
Source: J Biol Chem. 2026 Apr 1;302(5):111417. doi: 10.1016/j.jbc.2026.111417 (PMC13157077; doi:10.1016/j.jbc.2026.111417)
Supplement: Supplementary material [file mmc1.pdf]

Fortilin binds and stabilizes MEF2C, activates it through phosphorylation, and drives transcription of the cell structural and survival protein CTNNA3

Running title: Characterization of fortilin-MEF2C interaction

(RESEARCH ARTICLE TO JBC)

Mari Nakashima, Sandipan Mukherjee, Decha Pinkaew, Uttariya Pal, Jolanda van Hengel, Geert Berx, and Ken Fujise

SUPPLEMENTARY INFORMATION

Supplementary Figure Legends

Fig. S1 | RT-qPCR analysis of fortilin-competent and fortilin-deficient THP1 cells; Subcellular fractionation of 293T cells; in vitro pulldown assay evaluating the fortilin-MEF2C interaction. *Abbreviations:* WT, $THP1^{WT\text{-}fortilin}$ cells (THP1 cells expressing wild-type fortilin); KO, $THP1^{KO\text{-}fortilin}$ cells (THP1 cells in which the fortilin gene was deleted by Crispr-Cas9); AU arbitrary unit; VWF, von Willebrand factor; TF, tissue factor; IB, immunoblot; α-MEF2C, anti-MEF2C antibody (Ab); α-fortilin, anti-fortilin Ab; TCE, 2,2,2-trichloroethanol; α-FLAG, anti-FLAG (DYKDDDDK) antibody (Ab); α-His_6, anti-hexahistidine Ab; IP, immunoprecipitation; INPUT, 10% of total cell lysates used for IP; IgG, normal rabbit immunoglobulin G. **A–F.** RT-qPCR analysis of total RNA from $THP1^{WT\text{-}fortilin}$ and $THP1^{KO\text{-}fortilin}$ cells for fortilin (**A**), *CD68* (**B**), *MAC2* (**C**), *VWF* (**D**), *TF* (**E**), and *NOS3* (**F**) (n = 3; p values are provided for each gene analyzed; two-sample t test). **G.** In vitro co-IP assay. Recombinant human FLAG-tagged fortilin protein (fortilin-FLAG) was immunoprecipitated using α-fortilin Ab from a reaction mixture containing fortilin-FLAG, MEF2C-FLAG, and NQO2-His_6. The successful IP of fortilin-FLAG and the co-IP of MEF2C-FLAG were confirmed by western blotting of the eluates using α-FLAG Ab. **H.** Subcellular localization of MEF2C and fortilin. Cytoplasmic and nuclear fractions were isolated from 293T cells and analyzed by western blotting using α-MEF2C and α-fortilin Abs. Total protein loading was assessed by TCE staining.

Fig. S2 | Sequence analysis of MEF2A, MEF2B, MEF2C, and MEF2D.

A. Phylogenetic tree of MEF2A, MEF2B, MEF2C, and MEF2D. Scale bar represents phylogenetic evolutionary distance (unit: expected substitutions per site). **B.** Pairwise

amino acid identities among MEF2A, MEF2B, MEF2C, and MEF2D.

Fig. S3 | Time course-analysis of MEF2C degradation in the presence and absence of fortilin. *Abbreviations:* IB, immunoblot; α-FLAG, anti-FLAG antibody (Ab); α-fortilin, anti-fortilin Ab; MG132, carbobenzoxy-L-leucyl-L-leucyl-L-leucine (proteasome inhibitor); THP1$^{WT\text{-}fortilin}$, THP1 cells expressing wild-type fortilin (WT); THP1$^{KO\text{-}fortilin}$, THP1 cells in which the fortilin gene was deleted by Crispr-Cas9 (KO); TCL, total cell lysate; AU, arbitrary unit. Time-course analysis of MEF2C degradation in the presence or absence of MG132 using the JESS^{TM} capillary-based western blot system. THP1 cells were transiently transfected with pEZ-MEF2C^{WT}-FLAG, incubated for 48 h, treated with cycloheximide in the presence or absence of MG132 (defined as time 0), and harvested at the indicated time points. **A.** TCLs were analyzed by immunoblotting with α-FLAG and α-fortilin Abs to assess MEF2C^{WT}-FLAG and fortilin expression, respectively. Total protein loading was visualized using the JESS^{TM} total protein detection module. **B**–**D.** Quantitative analysis of MEF2C expression and degradation in THP1$^{WT\text{-}fortilin}$ and THP1$^{KO\text{-}fortilin}$ cells treated with and without MG132. Four experimental groups were analyzed—THP1$^{WT\text{-}fortilin}$ ± MG132 and THP1$^{KO\text{-}fortilin}$ ± MG132. MEF2C expression indices (AU) were calculated at each time point using JESS™ Compass software by dividing the area under the MEF2C peak by the total protein signal within the same capillary (“in-capillary normalization”). Absolute MEF2C expression levels at time 0 were first compared between THP1$^{WT\text{-}fortilin}$ and THP1$^{KO\text{-}fortilin}$ cells in the absence (**B**) and presence (**C**) of MG132. Data are presented as means ± standard deviation with P values indicated (n = 3, two-sample t test). MEF2C degradation kinetics were

then assessed by normalizing MEF2C expression indices at each time point to the corresponding time 0 value within each group and comparing degradation profiles between $THP1^{WT\text{-}fortilin}$ and $THP1^{KO\text{-}fortilin}$ cells in the absence and presence of MG132 (n = 3, two-sample t test) (**D**).

Fig. S4 | Fortilin promotes the phosphorylation of MEF2C at serine 59 in the N-terminal region. *Abbreviations:* IP, immunoprecipitation; SDS-PAGE, sodium dodecyl sulfate–polyacrylamide gel electrophoresis; p-MEF2C, phosphorylated MEF2C protein; IB, immunoblot; WT, $THP1^{WT\text{-}fortilin}$ (THP1 cells expressing wild-type fortilin); KO, $THP1^{KO\text{-}fortilin}$ (THP1 cells in which the fortilin gene was deleted by Crispr-Cas9); S^{59}, serine 59. **A.** Steps of phosphoprotein visualization. $THP1^{WT\text{-}fortilin}$ (WT) and $THP1^{KO\text{-}fortilin}$ (KO) cells were transiently transfected with a plasmid encoding FLAG-tagged MEF2C (MEF2C-FLAG)(**1**). MEF2C-FLAG was immunoprecipitated from total cell lysates (**2**) using anti-FLAG M2 magnetic beads (**3**), followed by extensive washing and acidic elution. Eluates were resolved by SDS–PAGE together with phosphoprotein molecular weight standards (**4**). Gels were fixed (**5**) and stained with Pro-Q Diamond to visualize phosphorylated MEF2C (**6**), and fluorescent signals were captured using the ChemiDoc MP Imaging System (Bio-Rad Laboratories)(**7**). The same gels were subsequently stained with SYPRO Ruby to detect total MEF2C protein (**8**), and images were acquired using UV transillumination (**9**). This sequential staining strategy enabled quantitative assessment of MEF2C phosphorylation relative to total MEF2C levels under fortilin-competent and fortilin-deficient conditions. Illustration created with BioRender.com. **B**, **C.** Evaluation of MEF2C phosphorylation at $serine^{59}$ in the presence and absence of

fortilin. Total cell lysates from THP1$^{WT\text{-}fortilin}$ (WT) and THP1$^{KO\text{-}fortilin}$ (KO) cells were probed on the JESS system. MEF2C expression was detected using an α-MEF2C antibody (**B**, left panel), and MEF2C phosphorylated at serine^{59} was detected using an α-phospho-serine^{59} MEF2C antibody (**B**, right panel). A phosphorylation index, defined as the ratio of MEF2C phosphorylated at serine^{59} to total MEF2C, was calculated and expressed in A.U. using JESS™ Compass software by dividing the area under the curve of the phosphorylated MEF2C peak by the total protein signal within the same capillary ("in-capillary normalization"). Data are presented as means ± standard deviation (*n* = 3, two-sample *t* test) (**C**).

Fig. S5 | Chromatin immunoprecipitation of CTNNA3 and the role of fortilin–MEF2C binding on CTNNA3 expression. *Abbreviations:* WT, THP1$^{WT\text{-}fortilin}$ (THP1 cells expressing wild-type fortilin); KO, THP1$^{KO\text{-}fortilin}$ (THP1 cells in which the fortilin gene was deleted by Crispr-Cas9; ChIP-qPCR, chromatin immunoprecipitation and quantitative PCR; chr, chromosome; hg38, nucleotide numbers according to the University of California, Santa Cruz (UCSC) Genome Browser; IP, immunoprecipitation; Pol II, RNA polymerase II; TSS, transcription start site. **A.** Steps of RNA polymerase II ChIP-qPCR assays. THP1$^{WT\text{-}fortilin}$ (WT) and THP1$^{KO\text{-}fortilin}$ (KO) cells were treated with 1% formaldehyde to crosslink protein–DNA complexes and quenched with glycine (**1**, **2**). Total cell lysates (TCLs) were generated, chromatin was sheared by sonication (**3**), and RNA polymerase II (Pol II) was immunoprecipitated using an anti–Pol II antibody (α-Pol II Ab) or normal IgG as a negative control, each coupled to Protein A/G beads (**4**). Beads were washed (**5**), and immunoprecipitated chromatin was eluted and reverse

cross linked (**6**). The recovered and purified DNA (**7**) was analyzed by quantitative PCR (qPCR) using primer sets flanking the transcription start sites (TSSs) of *CTNNA3*, *CD68*, and *VWF* (**8**, **9**). Pol II occupancy indices were calculated by normalizing the amount of TSS DNA fragments immunoprecipitated with the α–Pol II Ab to that of normal IgG and were expressed as arbitrary units (AU). Illustration created with BioRender.com. **B.** ChIP-qPCR target regions for *CTNNA3*, *CD68* (positive control), and *VWF* (negative control).

Fig. S1

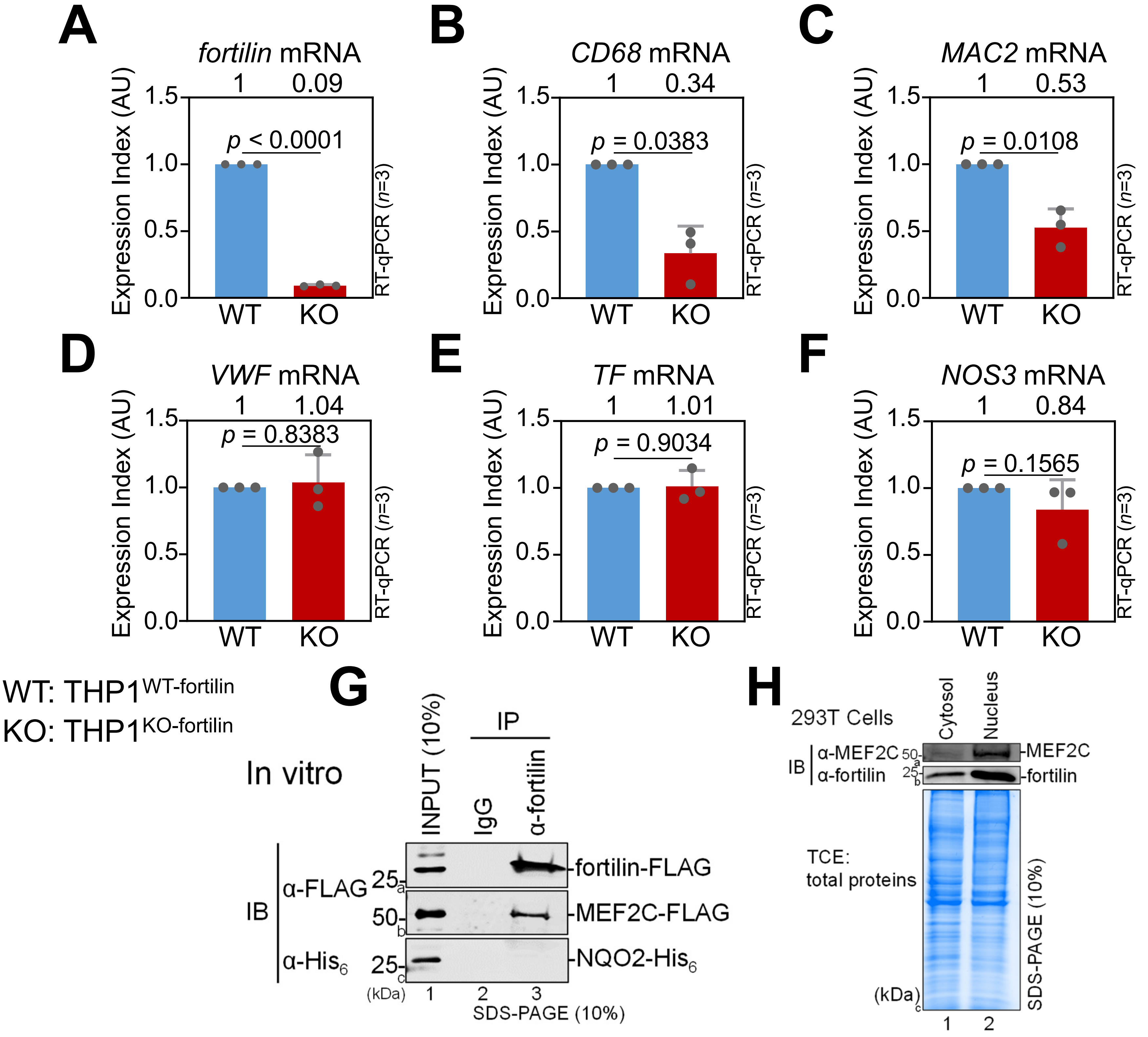

A
fortilin mRNA
1 0.09
p < 0.0001
Expression Index (AU)
RT-qPCR (n=3)
WT KO
B
CD68 mRNA
1 0.34
p = 0.0383
C
MAC2 mRNA
1 0.53
p = 0.0108
D
VWF mRNA
1 1.04
p = 0.8383
E
TF mRNA
1 1.01
p = 0.9034
F
NOS3 mRNA
1 0.84
p = 0.1565
WT: THP1WT-fortilin
KO: THP1KO-fortilin
G
In vitro
INPUT (10%)
IP
IgG
α-fortilin
IB
α-FLAG
α-His6
fortilin-FLAG
MEF2C-FLAG
NQO2-His6
(kDa)
SDS-PAGE (10%)
H
293T Cells
Cytosol
Nucleus
α-MEF2C
α-fortilin
MEF2C
fortilin
TCE:
total proteins
SDS-PAGE (10%)

Fig. S2

A

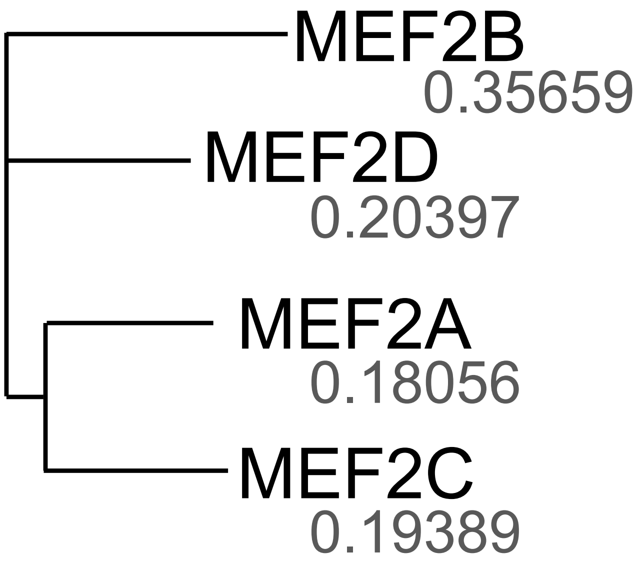

B

Sequence identity of MEF2 family

MEF2 family		**MEF2B**	**MEF2D**	**MEF2A**	**MEF2C**
Identity	**MEF2B**	100	43.94	40.4	41.31
	**MEF2D**		100	57.91	54.32
	**MEF2A**			100	62.56
	**MEF2C**				100
Amino Acid Length		368	521	497	463

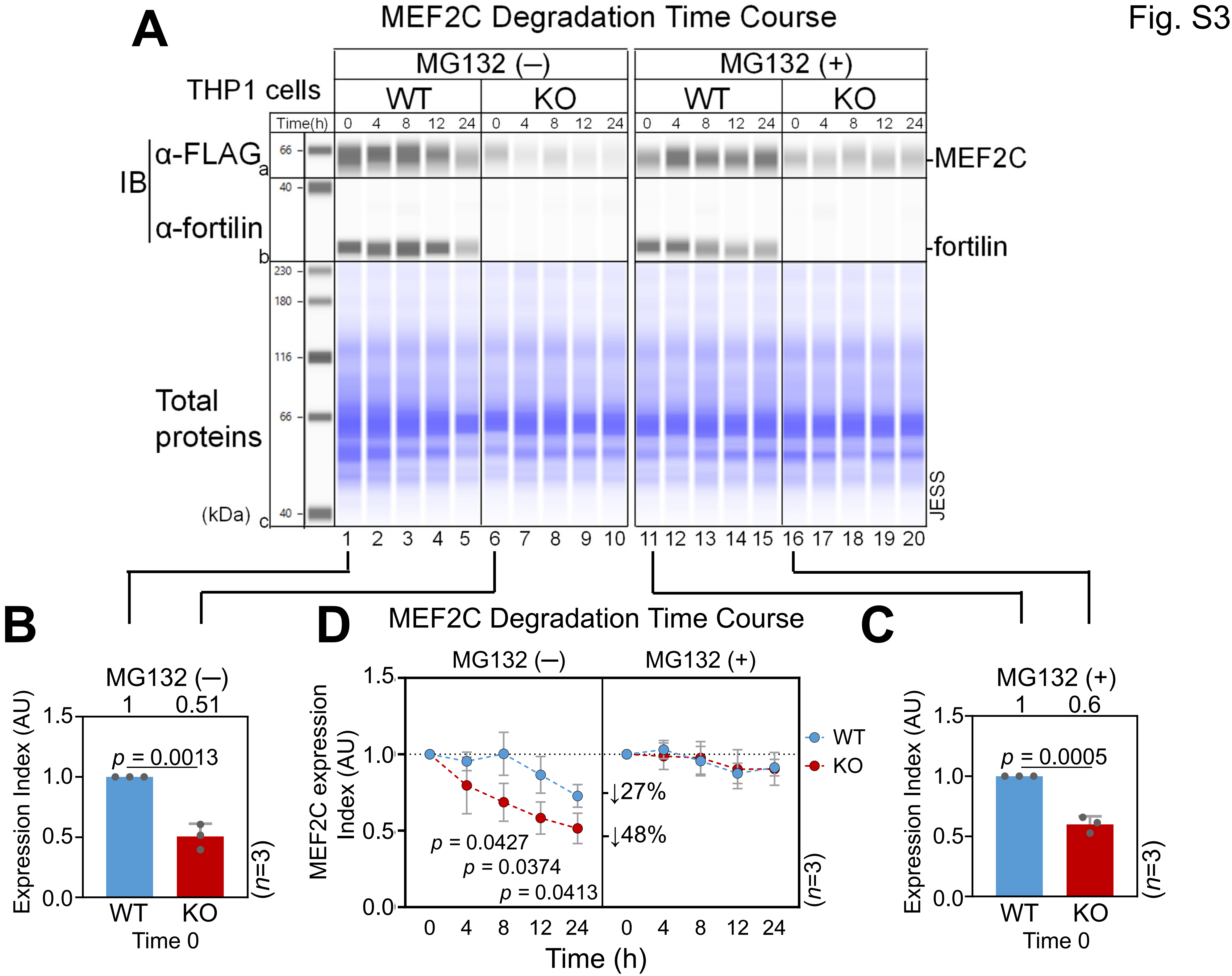

Fig. S3
A
MEF2C Degradation Time Course
THP1 cells
MG132 (–)
MG132 (+)
WT
KO
WT
KO
Time(h)
0 4 8 12 24 0 4 8 12 24 0 4 8 12 24 0 4 8 12 24
IB
α-FLAG
α-fortilin
MEF2C
fortilin
Total proteins
(kDa)
66
40
230
180
116
66
40
JESS
1 2 3 4 5 6 7 8 9 10 11 12 13 14 15 16 17 18 19 20
B
MG132 (–)
1 0.51
p = 0.0013
Expression Index (AU)
WT KO
Time 0
(n=3)
D
MEF2C Degradation Time Course
MG132 (–)
MG132 (+)
MEF2C expression Index (AU)
↓27%
↓48%
p = 0.0427
p = 0.0374
p = 0.0413
WT
KO
Time (h)
(n=3)
C
MG132 (+)
1 0.6
p = 0.0005
Expression Index (AU)
WT KO
Time 0
(n=3)

Fig. S4

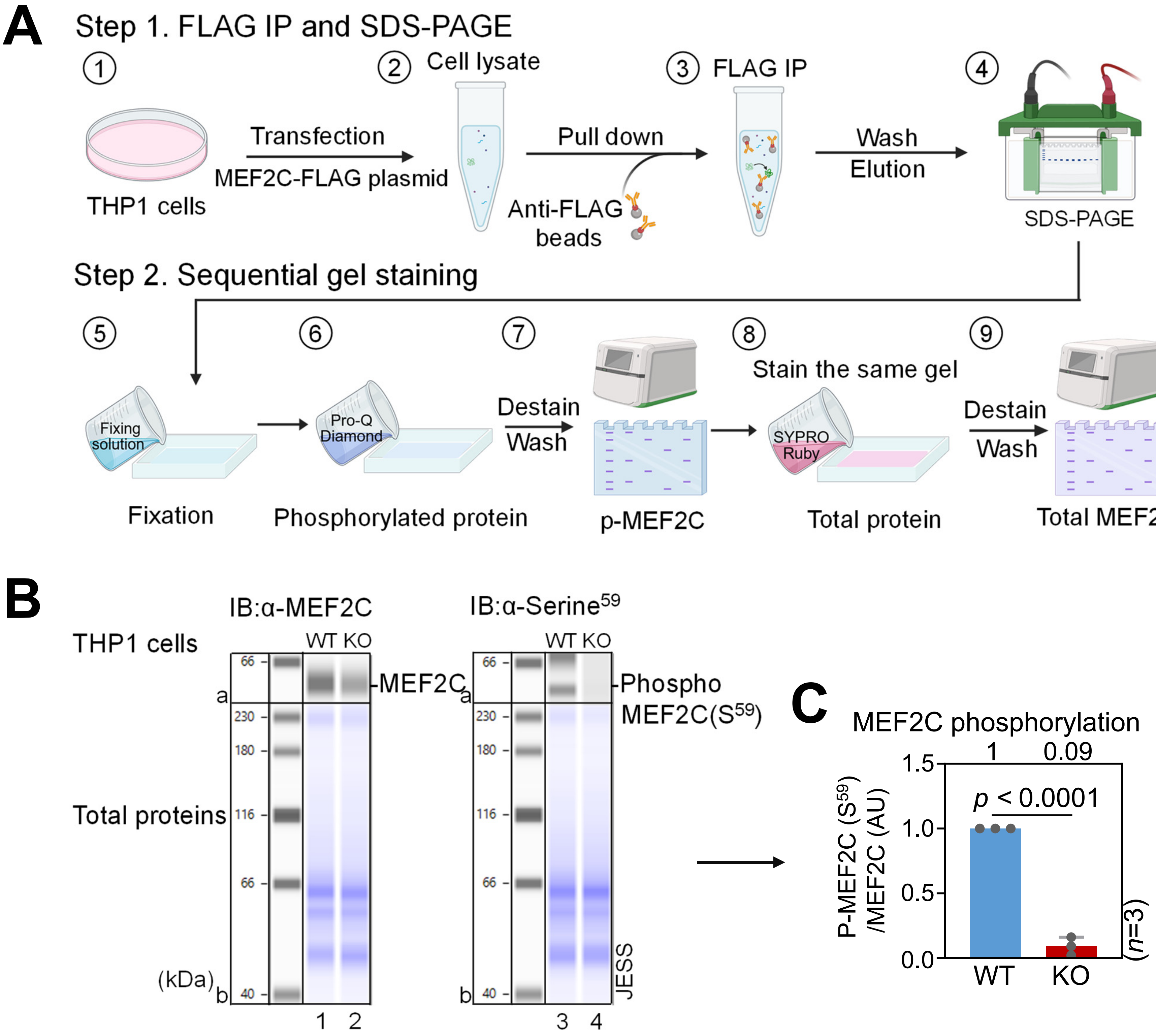
A
Step 1. FLAG IP and SDS-PAGE
1
2 Cell lysate
3 FLAG IP
4
Transfection
MEF2C-FLAG plasmid
THP1 cells
Pull down
Anti-FLAG beads
Wash
Elution
SDS-PAGE
Step 2. Sequential gel staining
5
6
7
8
9
Fixing solution
Pro-Q Diamond
Destain
Wash
Stain the same gel
SYPRO Ruby
Destain
Wash
Fixation
Phosphorylated protein
p-MEF2C
Total protein
Total MEF2C
B
IB:α-MEF2C
IB:α-Serine59
THP1 cells
WT KO
WT KO
MEF2C
Phospho MEF2C(S59)
a
a
Total proteins
(kDa)
b
b
66
230
180
116
66
40
JESS
1 2
3 4
C
MEF2C phosphorylation
1 0.09
p < 0.0001
P-MEF2C (S59) /MEF2C (AU)
1.5
1.0
0.5
0.0
WT
KO
(n=3)

Fig. S5

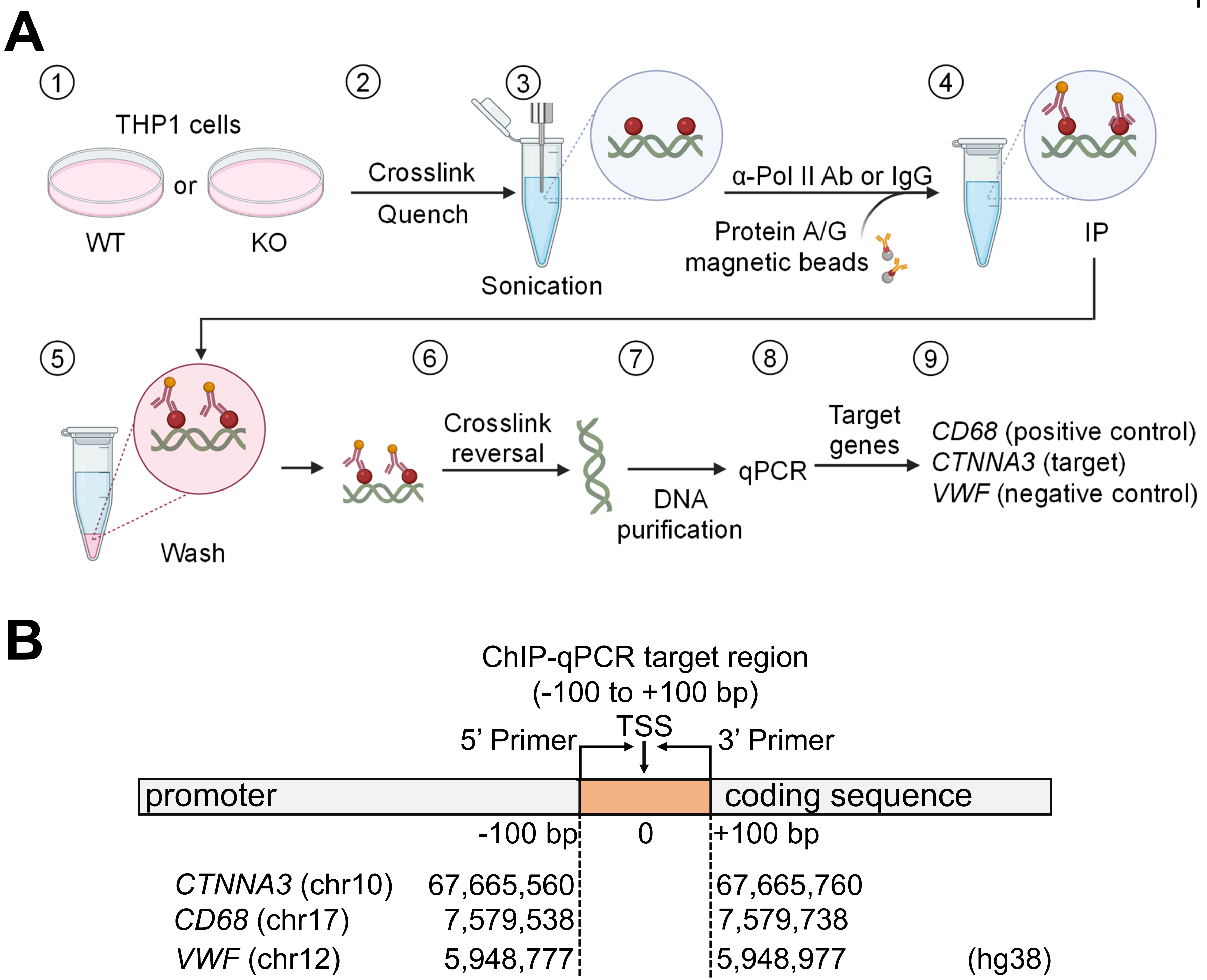
A
①
THP1 cells
or
WT
KO
②
Crosslink
Quench
③
Sonication
α-Pol II Ab or IgG
Protein A/G
magnetic beads
④
IP
⑤
Wash
⑥
Crosslink
reversal
⑦
DNA
purification
⑧
qPCR
⑨
Target
genes
CD68 (positive control)
CTNNA3 (target)
VWF (negative control)
B
ChIP-qPCR target region
(-100 to +100 bp)
TSS
5' Primer
3' Primer
promoter
coding sequence
-100 bp
0
+100 bp
CTNNA3 (chr10) 67,665,560 67,665,760
CD68 (chr17) 7,579,538 7,579,738
VWF (chr12) 5,948,777 5,948,977
(hg38)
